# Genetic variants related to physical activity or sedentary behaviour: a systematic review

**DOI:** 10.1186/s12966-020-01077-5

**Published:** 2021-01-22

**Authors:** Lene Aasdahl, Tom Ivar Lund Nilsen, Ingebrigt Meisingset, Anne Lovise Nordstoga, Kari Anne I. Evensen, Julie Paulsen, Paul Jarle Mork, Eivind Schjelderup Skarpsno

**Affiliations:** 1 Department of Public Health and Nursing, Faculty of Medicine and Health Sciences, Norwegian University of Science and Technology (NTNU), Postboks 8905, MTFS, 7491 Trondheim, Norway; 2 Unicare Helsefort Rehabilitation Centre, Rissa, Norway; 3 Clinic of Anaesthesia and Intensive Care, St. Olavs Hospital, Trondheim, Norway; 4 Department of Clinical and Molecular Medicine, NTNU, Trondheim, Norway; 5 Department of Physiotherapy, Oslo Metropolitan University, Oslo, Norway; 6 Unit for Physiotherapy Services, Trondheim, Norway; 7 Department of Medical Genetics, St. Olavs Hospital, Trondheim, Norway; 8 Department of Neurology and Clinical Neurophysiology, St. Olavs Hospital, Trondheim, Norway

**Keywords:** Exercise, Sedentary lifestyle, Alleles, Genetic association studies, Genetic markers, Genetic pleiotropy

## Abstract

**Background:**

Research shows that part of the variation in physical activity and sedentary behaviour may be explained by genetic factors. Identifying genetic variants associated with physical activity and sedentary behaviour can improve causal inference in physical activity research. The aim of this systematic review was to provide an updated overview of the evidence of genetic variants associated with physical activity or sedentary behaviour.

**Methods:**

We performed systematic literature searches in PubMed and Embase for studies published from 1990 to April 2020 using keywords relating to “physical activity”, “exercise”, “sedentariness” and “genetics”. Physical activity phenotypes were either based on self-report (e.g., questionnaires, diaries) or objective measures (e.g., accelerometry, pedometer). We considered original studies aiming to i) identify new genetic variants associated with physical activity or sedentary behaviour (i.e., genome wide association studies [GWAS]), or ii) assess the association between known genetic variants and physical activity or sedentary behaviour (i.e., candidate gene studies). Study selection, data extraction, and critical appraisal were carried out by independent researchers, and risk of bias and methodological quality was assessed for all included studies.

**Results:**

Fifty-four out of 5420 identified records met the inclusion criteria. Six of the included studies were GWAS, whereas 48 used a candidate gene approach. Only one GWAS and three candidate gene studies were considered high-quality. The six GWAS discovered up to 10 single nucleotide polymorphisms (SNPs) associated with physical activity or sedentariness that reached genome-wide significance. In total, the candidate gene studies reported 30 different genes that were associated (*p* < 0.05) with physical activity or sedentary behaviour. SNPs in or close to nine candidate genes were associated with physical activity or sedentary behaviour in more than one study.

**Conclusion:**

GWAS have reported up to 10 loci associated with physical activity or sedentary behaviour. Candidate gene studies have pointed to some interesting genetic variants, but few have been replicated. Our review highlights the need for high-quality GWAS in large population-based samples, and with objectively assessed phenotypes, in order to establish robust genetic instruments for physical activity and sedentary behaviour. Furthermore, consistent replications in GWAS are needed to improve credibility of genetic variants.

**Trial registration:**

Prospero CRD42019119456.

**Supplementary Information:**

The online version contains supplementary material available at 10.1186/s12966-020-01077-5.

## Background

Physical inactivity and sedentariness represent a major challenge to public health and contribute substantially to ill health and premature mortality [1, 2]. The impact of physical inactivity on development of non-communicable diseases has been compared to that of tobacco smoking, alcohol consumption, or an unhealthy diet [1, 3, 4]. In contrast, there is ample evidence that a physically active lifestyle is associated with a myriad of health benefits [5–9]. Despite this, a large proportion of the population remains inactive below the recommended levels of physical activity [10]. Although the variation in physical activity and sedentariness is likely to be determined by a multitude of factors, evidence from family- and twin studies suggest a significant genetic influence [11, 12].

Recent developments in both objective measurements of physical activity and sedentary behaviour [13, 14], along with improved genotyping technology facilitating extensive genotyping in large populations [15], give promise for the identification of valid and robust genotype-phenotype associations of physical activity and sedentary behaviour. These associations may in turn serve as genetic instruments in Mendelian randomisation studies [16] to improve causal inference about the health effects of physical activity and sedentariness [17], and thus guide the development of effective preventive strategies and interventions.

Previous reviews have reported associations between different physical activity and sedentary behavior phenotypes and various genes [12, 18–20]. However, most reviews did not describe a systematic literature search [18–20] and no previous review has conducted a quality assessment to critically assess the methodological quality of the included studies, which is recommended for systematic reviews of genetic association studies [21]. The aim of the current systematic review was therefore to provide a comprehensive overview of genetic variants associated with physical activity or sedentary behaviour.

## Methods

The review protocol was registered in Prospero (International prospective register of systematic reviews): CRD42019119456. The results are presented according to the PRISMA statement [22].

### Eligibility criteria

We included all original studies on humans of any age, published in English in international peer-review journals, that 1) identified new genetic variants associated with physical activity or sedentary behaviour (i.e., GWAS), or 2) reported the association between a genetic variant and these behaviours (i.e., candidate gene studies). Studies assessing physical activity or sedentary behaviour as a modifier/moderator of genetic variants associated with other outcomes were not included. We did not include case reports, editorials or reviews, or studies solely including animals.

The phenotype definitions of physical activity and/or sedentary behaviour in the included studies were defined based on data from self-reports (e.g. questionnaires, diaries) or objective measurements (e.g. accelerometry, pedometer). We excluded studies that only measured fitness or strength, or with an aim to study genes associated with performance in sports. Furthermore, we excluded studies that only reported on physical activity related to active transport or occupational activity. Studies using a polygenic risk score (i.e. not reporting associations for individual genetic variants), or studies examining interaction were excluded if no estimate on the association between genetic variants and physical activity or sedentary behaviour was reported.

### Information sources and search strategy

Studies were identified by searching electronic databases and inspecting reference lists of studies and relevant systematic reviews. The design and execution of the literature search were supervised by a trained research librarian with expertise in systematic reviews. The search was performed in PubMed and Embase (via Ovid) from 1990 until April 14th 2020. The search strategy was based on domains related to physical activity, sedentary activity and genetics. The full search strategy is presented in online supplementary 1.

### Study selection

**Fig. 1:**
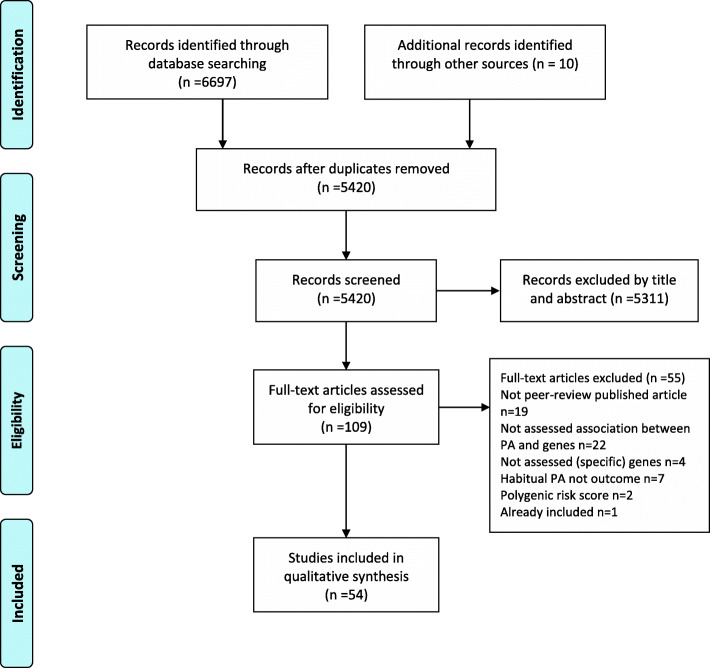
Flowchart for the selection of studies

Eligibility assessment was performed in a two-stage screening process, described in Bramer et al. [23]. In the first stage, titles and abstracts were screened by three pairs of two researchers (ALN/ESS, IM/KAIE, TILN/LA) blinded to each other’s selection. These pairs remained the same throughout all the steps of the review process. Disagreements within pairs were discussed and resolved by a third researcher (PJM) when necessary. Studies considered not to be relevant were excluded and full-text articles were obtained for the remaining studies. In the second stage, two reviewers independently screened the full-text articles against the inclusion and exclusion criteria. If necessary, disagreements were resolved by discussion with a third reviewer. Reasons for excluding studies were recorded (Fig. 1).

### Data extraction

We developed a data extraction form (online supplementary 2) inspired by Eskola et al. [24]. The form was adopted to the purpose of the current study and pilot-tested. Two researchers extracted data independently using the form. Disagreements were resolved by discussion between the two reviewers and if necessary, discussed with a third researcher (PJM). The following data was extracted from the included studies, if available: 1) general information (authors and year of publication); 2) participant characteristics (country of origin, ethnicity, age and gender); 3) study characteristics (study design, genotyping method, and physical activity measuring instrument); 4) outcomes/results (physical activity phenotype, genetic variant, strength of association, confidence interval and/or *p*-value).

### Risk of bias and methodological quality

We developed criteria for assessing risk of bias and methodological quality of the included studies (online supplementary 2). The criteria were inspired by Hayden et al. [25] and Eskola et al. [24] and assessed the following: selection bias (inclusion/exclusion criteria and population stratification), sample size calculations, genetic data (DNA sampling, genotyping method, quality control, blinding and Hardy-Weinberg equilibrium), physical activity and sedentary behaviour data (assessment procedure, validation and whether self-reported or objectively measured) and statistical analyses (measure of association and replication within the study), giving a maximum score of 12 points. The studies where then classified according to their score value: very low quality, < 3 points; low quality, 3–5.5 points; medium quality 6–8.5 points; high quality, ≥9 points. Three pairs of researchers (ALN/ESS, IM/KAIE, TILN/LA) assessed the criteria independently.

### Data synthesis and analysis

Due to the expected heterogeneity of phenotypes and genetic markers, we did not aim for a quantitative data synthesis involving a meta-analyses approach. The results of the individual studies are presented and discussed according to their scores on the risk of bias and quality assessment, putting more emphasis on studies with a higher quality score. For the genetic variants identified in candidate gene studies, we also report their association with accelerometry defined phenotypes using summary statistics [26] from the high quality GWAS by Doherty et al. [27].

Most candidate gene studies only presented results that had a *p*-value < 0.05 (i.e., nominally statistically significant). To avoid bias in the extracted results from the different studies, we only retrieved associations that had a *p*-value < 0.05 from studies that also reported results with higher *p*-values.

## Results

### Search results and selection of studies

Figure 1 shows a PRISMA flowchart of the study selection process. In total, 6697 records were identified through the database search and 10 records through inspection of reference lists or citation tracking. After removal of 1287 duplicates, 5420 records were screened at title and/or abstract level and 109 full-text articles were assessed for eligibility. Of these, 54 articles were found eligible for inclusion in the current review (online supplementary 3).

### Characteristics of the included studies

Table 1 shows the main characteristics of the included studies. Among the included studies 48 used a candidate gene approach and six were GWAS, where three also examined candidate genes. The phenotypes of physical activity and sedentary behaviour were operationalized in a variety of ways and mainly measured by questionnaires. In total, 12 studies used objectively measured physical activity data (accelerometry) of which two were GWAS.

**Table 1:** Characteristics of the included studies in alphabetical order, grouped by study type (i.e., GWAS and candidate gene studies)

1st author (year)	Population	Ethnicity^**a**^	Sample size, n (% female)	Age, years - mean (SD) - median - range	GWAS/ candidate gene	Measurement instrument(s)
**GWAS**						
De Moor (2009) [28]	General (twins)	Two groups Caucasian +N/R	2755 (59%) Total for both groups	43.5 (14.6)/50.0 (18.3)^b^ N/R 14.5–79.8/19.1–87.2^b^	GWAS + candidate genes (*ACE, CASR, CYP19A1, DRD2, LEPR, MC4R*)	Questionnaire (name N/R)
Doherty (2018) [27]	General	Caucasian	91,105 (56%)	N/R N/R 45–80	GWAS	Accelerometry (wrist)
Hara (2018) [29]	General	Japanese	13,980 (55%) +replication	54.8 (9.4) N/R N/R	GWAS + candidate genes (*DNAPTP6, PAPSS2, C18orf2, GABRG3, LEPR, RN7SK–SLC44A1*)	Questionnaire (IPAQ-s)
Kim (2014) [75]	General	N/R	8454 (53%)	52.2 (8.9) N/R 40–69	GWAS	Questionnaire (name N/R)
Klimentidis (2018) [30]	General	Caucasian	377,234 (N/R) +replication	N/R N/R N/R	GWAS	Questionnaire (IPAQ-l) Subgroup (*n* = 91,084) accelerometry (wrist)
Lin (2018) [31]	General (three separate studies)	Main study: African American + Caucasian	11,865 (100%)	N/R N/R 50–79	GWAS + candidate genes (*LEPR, CASR, PAPSS2, DRD2, GABRG3, CYP19A1, ACE, MC4R*)	Questionnaires (40-item Jackson Heart Physical Activity Cohort survey) + other questionnaires (name N/R)
**Candidate gene studies**						
Adamska-Patruno (2019) [76]	General	Caucasian	927 (49%)	40.2 (0.5)^c^ N/R N/R	*MC4R*	Questionnaire (IPAQ-l)
Berentzen (2008) [77]	General population (males)	Caucasian	551 (0%)	- N/R - 47.0/49.5 (obese/controls)^b^ - N/R	*FTO*	Questionnaire (name N/R)
Boer (1997) [78]	General (students)	N/R	1994 (N/R)	N/R N/R 18–26	*ApoE*	Questionnaire (name N/R)
Bruneau (2017) [32]	General (adults)	Caucasian	461 (56%)	24.1 (15.7) N/R N/R	*ACE*	Questionnaire (PPAQ)
Bruneau (2018) [33]	General (adults)	Caucasian	532 (55%)	23.4 (0.2) N/R N/R	*IL-15*	Questionnaire (PPAQ)
Cole (2010) [34]	General (families with obese children)	Hispanic	1030 (50%)	11.0 (3.9) N/R N/R	*MC4R*	Accelerometry (wrist)
Camps (2019) [35]	General	N/R	148 (74%)	42 (9) N/R 18–50	*ADRB2, FTO, MC4R, PPARG2, PPARD, PPARGC1A*	Accelerometry (waist)
Espinosa-Salinas (2019) [79]	General	Caucasian	451 (N/R)	N/R N/R 18–65	64 different genetic variants	Questionnaire (MLTPAQ + frequency question)
Flack (2019) [36]	General	N/R	178 (71%)	26.9 (8.6) N/R 18–49	*ACE, TPH2, CNR1, DRD3, FTO, HTR2A, DRD2/ANKK1, PAPSS2, LEPR, GABRG3, BDNF, DRD2, COMT, DRD1, DRD4, DBH*	Accelerometry (hip)
Fonseca-Portilla (2019) [80]	General	Latino and Mexican	349 (67%)	42 (N/R) - N/R - N/R	*BNDF*	Questionnaire (name N/R)
Fuentes (2002) [81]	General (adults)	Caucasian	455 (63%)	44 (N/R)/45 (N/R)^b^ N/R N/R	*ACE*	Questionnaire (name N/R)
Gielen (2014) [37]	General (twins)	Caucasian	222 (57%)	21 (2)/22 (5)^b^ N/R N/R	*PPARD, PPARGC1A, NRF1, MTOR*	Accelerometry (lower back)
Goleva-Fjellet (2020) [82]	General	Norwegian or Scandinavian	831 (50%)	55.5 (3.8) N/R N/R	*ACTN3, ACE, MAOA*	Questionnaire (name N/R)
Good (2015) [83]	General (children)	Diverse	651 (54%)	DNA-samples (15 years old), physical activity level (4.5 years old)	*MAOA*	Questionnaire (child behaviour questionnaire)
Grady (2013) [84]	General (elderly)	Caucasian (98%)	310 (71%)	95.2 (N/R) N/R 90–109	*DRD4*	Questionnaire (name N/R)
Haber (2010) [85]	General (men)	N/R	387 (0%)	47 (12.8) N/R 23–72	5-HT serotonin receptor	Questionnaire (IPAQ, version N/R)
Hakanen (2009) [38]	General (children)	N/R	438 (N/R)	All 15 years old	*FTO*	Questionnaire (name N/R)
Harbron (2014) [86]	General	Caucasian	133 (84%)	32.9 (4.4) N/R N/R	*FTO*	Questionnaire (Baecke Habitual Physical Activity Questionnaire)
Hubacek (2011) [87]	General	Caucasian	6024 (54%)	58.1 (6.9) N/R 45–69	*FTO*	Questionnaire (name N/R)
Huppertz (2014) [88]	General (twins)	Dutch/Western European	8768 (62%)	32.5 (12.3) N/R 7–50	*DRD1–5* *DBH* *COMT* *DAT1*	Questionnaire (name N/R)
Jensen (2014) [89]	General (children)	N/R	268 (52%)	N/R N/R N/R	*FADS*	Accelerometer (hip)
Jozkow (2009) [90]	General population (adult men)	Caucasian	360 (0%)	47 (12) N/R 24–72	Androgen receptor	Questionnaire (IPAQ, version N/R)
Jozkow (2011) [91]	General population (adult men)	Caucasian	311 (0%)	47 (12) N/R 24–72	*MC4R*	Questionnaire (IPAQ version N/R)
Jozkow (2013) [92]	General population (adult men)	Caucasian	397 (0%)	47 (12) N/R 24–72	*DRD2* *DRD4*	Questionnaire (IPAQ-l)
Kirac (2016) [93]	General (obese and healthy)	N/R	200 (N/R)	33.7 (9.4)/27.8 (8.3)^b^ N/R N/R	*MC4R* *FTO* *NMB*	Accelerometry (hip)
Klimentidis (2016) [39]	General	Caucasian (+Hispanic Americans and African-Americans in replication cohort)	7318 (52%)	45.4 (10.9) N/R N/R	*FTO*	Questionnaire (name N/R)
Lee (2015) [40]	General	Caucasian	492 (53%)	23.5 (0.3) N/R N/R	*FTO, KCTD15, MC4R, NEGR1, SH2B1, TMEM18*	Questionnaire (PPAQ)
Liu (2010) [41]	General (youth)	Caucasian and African American	750 (46%/56%)^b^	N/R N/R 8–12/14-18^b^	*FTO*	Questionnaire (name N/R), Accelerometry (*n* = 525, placement N/R)
Loos (2005) [42]	General (families)	French-Canadian	669 (55%)	52 (3.4)/28 (8.7)^b^ N/R N/R	*MC4R, MC3R, NPY* *NPY Y1R, CART, AGRP, POMC*	Questionnaire (name N/R) + diary (3-days)
Lorentzon (2001) [94]	General (adolescent girls)	Caucasian	97 (100%)	16.9 (1.2) N/R N/R	*CASR*	Questionnaire (name N/R)
Luglio (2016) [95]	Obese female adolescents	N/R	78 (100%)	13.7 (0.9) N/R 13–15	*UCP2, UCP3*	Questionnaire (name N/R)
Maestu (2013) [43]	General (adolescents)	N/R	261 (0%)	12.0 (0.8)	*ACE*	Accelerometry (hip)
Many (2017) [44]	General (students)	Mainly Caucasian (77%)	288 (52%)	22.4 (2.8) N/R 18–35	*ACTN3, ARDB1, ADRB3*	Questionnaire (PPAQ) + single questions
Moleres (2009) [96]	General (adolescents)	N/R	504 (N/R)	14.5 (1.1)/14.6 (1.1)^b^ N/R 13–18	*IL6*	Questionnaire (name N/R)
Murakami (2014) [45]	General	Japanese	556 (73%)	47.8 (8.0)/49.3 (10.9)/50.5 (9.7)^b^ N/R 24–65	*LEPR*	Accelerometer (lower back)
Murakami (2017) [46]	General	Japanese	648 (74%)	Women 53.7 (11.0) Men 49.4 (12.5) N/R 26–82	*DRD2/ANKK1*	Accelerometry (waist) + questionnaire (name N/R)
Reddon (2016) [47]	General (high risk for diabetes mellitus typer 2)	Multiple	9228 (N/R)	N/R N/R 18–85	14 different genes	Questionnaire (name N/R)
Richert (2007) [48]	General (adolescents)	Caucasian	222 (0%)	7.4 (0.4) N/R N/R	*LEPR*	Questionnaire (name N/R)
Salmén (2003) [97]	General (early postmenopausal women)	N/R (Finnish)	331 (100%)	52.7 (2.3) N/R N/R	*CYP19*	Single question
Simonen (2003) [98]	General (family study)	French- Canadian	721 (56%)	Women 40.1 (14.2) Men 41.2 (15.3)	*DRD2*	Questionnaire (name N/R) + diary (3-days)
Van der Mee (2018) [99]	General (twins)	Western European	12,929 (60%)	32.5 (16.0) N/R 12–90	*DRD1, DRD2/ANKK1, DRD3, DBH, COMT, DAT1, DRD4, DRD5, MAOA*	Questionnaire (N/R)
Van Deveire (2012) [49]	General	Caucasian	536 (55%)	23.4 (0.2) N/R N/R	*ANKRD6*	Questionnaire (PPAQ)
Vimaleswaran (2010) [100]	General (children and adolescents)	N/R	2062 (54%)	9.6 (0.4)/15.5 (0.5)^b^ N/R 8.4–11.3/14.1–17.8^b^	*PCK1*	Accelerometry (placement N/R) + single question
Walsh (2012) [101]	General (sedentary)	Caucasian	242 (54%)	23.4 (5.4) N/R 18–39	*LEP19*	Questionnaire (PPAQ)
West (2018) [102]	General	Caucasian	408 (20%)	34.9 (9.5) N/R N/R	*FTO*	Questionnaire (IPAQ-s)
Wilkinson (2013) [50]	General (adolescents)	Mexican and Mexican-American	1130 (51%)	- 14.3 (1.0) - N/R - N/R	*SNAP25, CNR1, TPH2* *ACE*	Two questions from the Youth Risk Behavioural Surveillance System
Winnicki (2004) [51]	Patients (untreated hypertension)	Caucasian	355 (25%)	33 (9) N/R N/R	*ACE*	Questionnaire (name N/R)
Wong (2012) [52]	Hospital employees	Chinese (in Singapore)	110 (65%)	32.7 (11.2) N/R 21–61	*ACE*	Questionnaire (IPAQ-s)

*Abbreviations*: *GWAS* genome-wide association study, *IPAQ-l* International Physical Activity Questionnaire long form, *IPAQ-s* International Physical Activity Questionnaire short form, *MLTPAQ* Minnesota Leisure Time Physical Activity Questionnaire, *N/R* not reported, *PPAQ* Paffenbarger Physical Activity Questionnaire, *SD* standard deviation

^a^ Ethnicity: white, European, American European and European descent are reported as Caucasian

^b^ Numbers only reported for the different groups, not for total sample

^c^ Most likely standard error, not specified

### Critical appraisal

Risk of bias and methodological quality varied considerable between the included studies (Table 2). The scores for the GWAS ranged from 7 to 9 with a median of 7.75. One GWAS was considered high quality [27]. Among the 48 candidate gene studies, the scores varied from 1 to 9.5, with a median of 6.5. Three candidate gene studies were considered high quality [32, 33, 41], 35 medium quality, while 10 studies were considered low or very low quality.

**Table 2:** Quality/risk of bias assessment for the included GWAS and candidate gene studies. Studies sorted in descending order according to quality score (high to low)

1st Author (year)	Incl./ excl.	Pop. strat.	Sample/ power	DNA sampl.	Genotyping			Hardy-Weinb.	Phenotype definition			Strength assoc.^**a**^	Replicated^**a**^	Sum (0–12)
					Method descr.	Quality control	Blinded		Assesment descr.	Valid. instr.	Object. measure			
**GWAS**														
Doherty (2018) [27]	1	1	0	1	1	1	0	1	1	1	1	N/A	0	9
Klimentidis (2018) [30]	0.5	1	0	0	1	1	0	1	1	1	1	N/A	1	8.5
Hara (2018) [29]	1	1	0	1	1	1	0	1	1	0	0	(1)^b^	1	8
De Moor (2009) [28]	0.5	1	0	1	1	1	0	1	1	0	0	(0)^b^	1	7.5
Kim (2014) [75]	1	0	0	1	1	1	0	1	1	0	0	N/A	1 (bootstrapping)	7
Lin (2018) [31]	0	1	0	0	1	1	0	1	1	1	0	(1)^b^	1	7
**Candidate gene studies**														
Bruneau (2017) [32]	1	0.5	1	1	1	1	0	1	1	1	0	1	N/A	9.5
Bruneau (2018) [33]	1	0.5	1	1	1	1	0	1	1	1	0	1	N/A	9.5
Liu (2010) [41]	1	0.5	0	1	1	1	0	1	1	1	1	1	N/A	9.5
Gielen (2014) [37]	1	0.5	0	1	1	0	0	1	1	1	1	1	N/A	8.5
Jozkow (2011) [91]	1	0.5	0	1	1	0	1	1	1	1	0	1	N/A	8.5
Jozkow (2013) [92]	1	0.5	0	1	1	0	1	1	1	1	0	1	N/A	8.5
Murakami (2014) [45]	1	0.5	0	1	1	0	0	1	1	1	1	1	N/A	8.5
West (2018) [102]	1	0.5	1	1	1	0	0	1	1	1	0	1	N/A	8.5
Camps (2019) [35]	1	0	0	1	1	0	0	1	1	1	1	1	N/A	8
Maestu (2013) [43]	1	0	0	1	1	0	0	1	1	1	1	1	N/A	8
Van der Mee (2018) [99]	1	1	0	0	1	1	0	1	1	1	0	1	N/A	8
Cole (2010) [34]	1	0.5	0	1	1	1	0	1	1	0	1	0	N/A	7.5
Vimaleswaran (2010) [100]	0.5	0	1	0	1	0	0	1	1	1	1	1	N/A	7.5
Wong (2012) [52]	1	0.5	0	1	1	0	0	1	1	1	0	1	N/A	7.5
Espinosa- Salinas (2019) [79]	1	0	0	1	1	0	0	1	1	1	0	1	N/A	7
Flack (2019) [36]	1	0	0	1	1	0	0	0	1	1	1	1	N/A	7
Goleva-Fjellet (2020) [82]	0.5	0.5	0	1	1	1	0	1	1	0	0	1	N/A	7
Huppertz (2014) [88]	1	1	1	0	0	1	0	1	1	0	0	1	N/A	7
Lee (2015) [40]	1	0	0	1	1	0	0	1	1	1	0	1	N/A	7
Many (2017) [44]	1	0	0	1	1	0	0	1	1	1	0	1	N/A	7
Reddon (2016) [47]	1	1	1	0	1	0	0	1	1	0	0	1	N/A	7
Van Deveire (2012) [49]	0.5	0.5	0	1	1	0	0	1	1	1	0	1	N/A	7
Adamska- Patruno (2019) [76]	0.5	0	0	1	1	0	0	1	1	1	0	1	N/A	6.5
Fonseca-Portilla (2019) [80]	1	0.5	1	1	1	0	0	0	1	0	0	1	N/A	6.5
Haber (2010) [85]	0	0.5	0	1	1	0	0	1	1	1	0	1	N/A	6.5
Hakanen (2009) [38]	0.5	0	0	1	1	1	0	1	1	0	0	1	N/A	6.5
Harbron (2018) [86]	1	0.5	0	1	1	0	0	1	1	1	0	0	N/A	6.5
Hubacek (2011) [87]	1	0.5	0	1	1	0	0	1	1	0	0	1	N/A	6.5
Jozkow (2009) [90]	1	0.5	0	1	1	0	0	0	1	1	0	1	N/A	6.5
Lorentzon (2001) [94]	1	0.5	0	1	1	0	0	1	1	0	0	1	N/A	6.5
Moleres (2009) [96]	0.5	0	0	1	1	1	0	1	1	0	0	1	N/A	6.5
Richert (2007) [48]	1	0.5	0	1	1	0	0	1	1	0	0	1	N/A	6.5
Walsh (2012) [101]	1	0.5	0	0	1	1	0	1	1	0	0	1	N/A	6.5
Jensen (2014) [89]	1	0	0	1	1	1	0	0	1	0	1	0	N/A	6
Kirac (2016) [93]	1	0	0	1	1	0	0	0	1	0	1	1	N/A	6
Klimentidis (2016) [39]	0.5	0.5	1	1	1	0	0	0	1	0	0	1	N/A	6
Loos (2005) [42]	0.5	0.5	0	1	1	0	0	1	1	0	0	1	N/A	6
Luglio (2016) [95]	1	0	0	1	1	0	0	1	1	0	0	1	N/A	6
Berentzen (2008) [77]	1	0.5	0	0^a^	1	0	0	1	1	0	0	1	N/A	5,5
Fuentes (2002) [81]	1	0.5	0	1	0	0	0	1	1	0	0	1	N/A	5.5
Murakami (2017) [46]	1	0.5	0	0	1	0	0	1	1	0	0^c^	1	N/A	5.5
Wilkinson (2013) [50]	0.5	1	0	1	0	0	0	1	1	0	0	1	N/A	5.5
Simonen (2003) [98]	0.5	0.5	0	0	1	0	0	1	1	0	0	1	(1)^d^	5
Winnicki (2004) [51]	0.5	0.5	0	0	1	0	0	1	1	1	0	0	N/A	5
Boer (1997) [78]	1	0	0	1	0	0	0	0	1	1	0	0	N/A	4
Grady (2013) [84]	0.5	0.5	0	1	0	0	0	1	0	0	0	1	N/A	4
Salmen (2003) [97]	1	0	0	1	1	0	0	0	0	0	0	1	N/A	4
Good (2015) [83]	0	0	0	0	0	0	0	0	1	0	0	0	N/A	1

^a^ Strength of association intended for candidate genes studies and replication for GWASs

^b^ The study also examines candidate genes, but this item is not included in the total score

^c^ Some analyses included objective data on physical activity, but not those reported here

^d^ The study included a replication cohort, but this item is not included in the total score

Few studies described a priori sample size calculation and only two studies described blinded genotyping. Most GWAS scored high on description of the genotyping process and phenotype definition, but only three studies used a validated self-reported instrument or objective measurement of physical activity or sedentary behaviour. Most of the candidate gene studies had a limited description about the quality control for the genotyping process.

### Associations between genes and habitual physical behaviour

The characteristics of the included studies (Table 1) and the risk of bias assessment (Table 2) are presented according to type of study (i.e., GWAS or candidate gene study). Table 3 shows the results for medium and high-quality studies ordered by chromosome. For a more detailed overview for all included studies see online supplementary 4 (GWAS) and 5 (candidate gene studies).

**Table 3:**
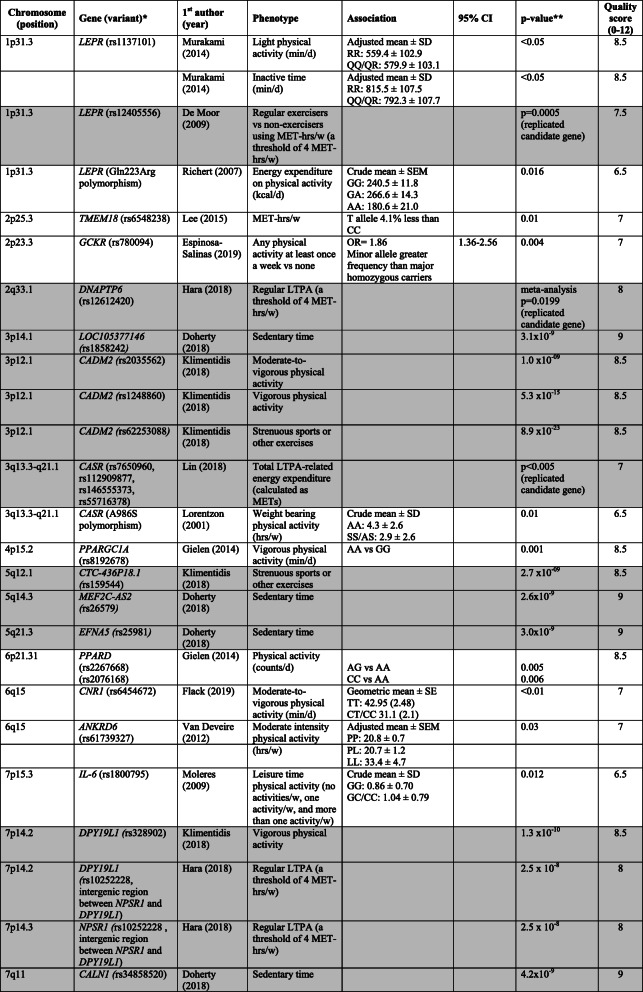

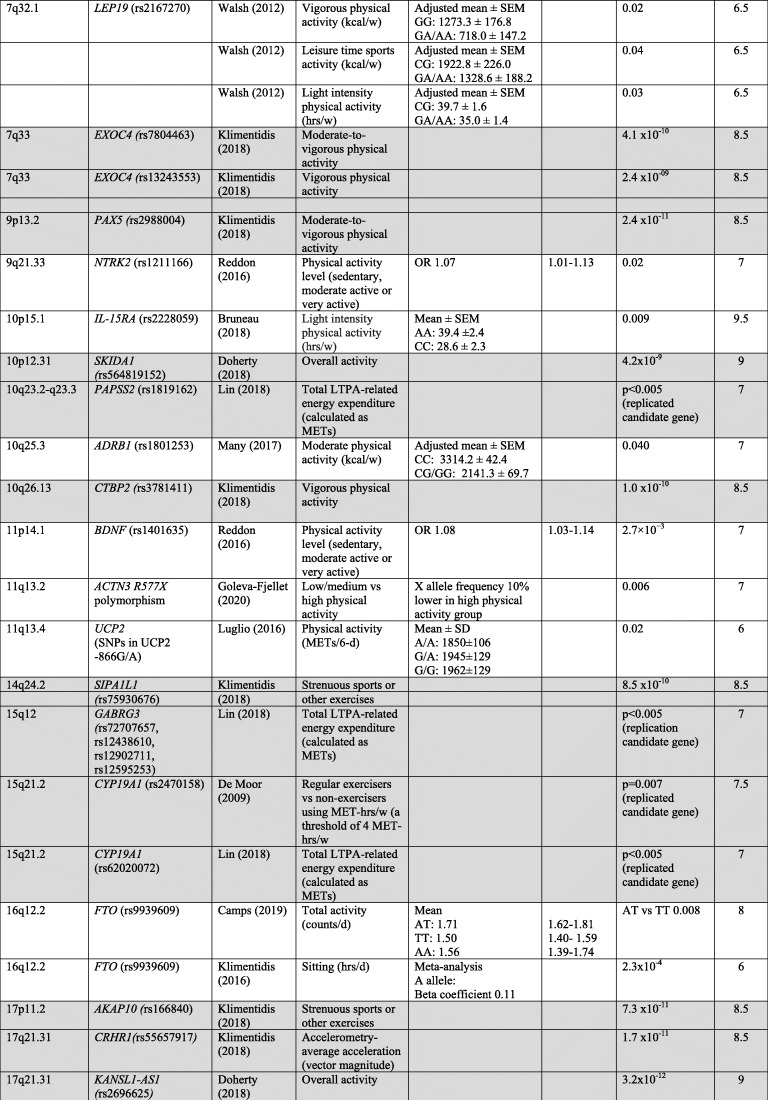

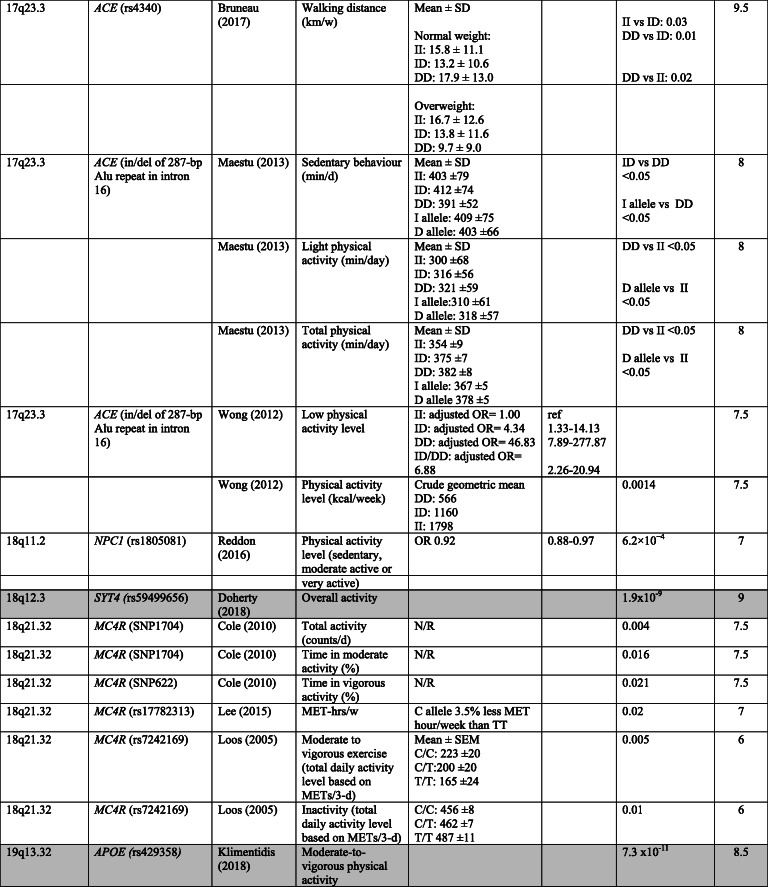
Genotype-phenotype associations in medium (6–8.5 points) and high (≥9 points) quality candidate gene studies and GWAS. For GWAS, results with a genome-wide significance level of *p* < 5 × 10^−8^ or lower are presented. GWAS are indicated by grey cells. Studies are sorted according to chromosome position

*Abbreviations*: *d* day, *hrs* hours, *in/del* insertion/deletion, *LTPA* leisure time physical activity, *MET* metabolic equivalent, *OR* odds ratio, *SNP* single nucleotide polymorphism, *VNTR* variable number tandem repeat, *w* week, *y* year

*For GWAS genes reported to be closest to the SNP is presented

**For GWAS: *p*-value SNP in discovery cohort

#### GWAS

In the six included GWAS, several SNPs were identified that were associated with physical activity or sedentary behaviour (Table 3 and online supplementary 4). Three studies [27, 29, 30] used a genome-wide significance level of *p* < 5 × 10^− 8^ or lower. The high-quality GWAS [27] was based on data from the UK Biobank and identified three loci associated with overall physical activity and four loci associated with sedentary behaviour. Also based on data from the UK Biobank, Klimentidis et al. [30] identified 10 loci that were associated with at least one of four physical activity phenotypes (i.e., moderate-to-vigorous physical activity, vigorous physical activity, strenuous sport or other exercises and overall physical activity level assessed by accelerometry). SNPs in *CADM2* were associated with all three phenotypes, whereas SNPs in *EXOC4* were associated with the first two. One SNP in *DPY19L1* was associated with vigorous physical activity only. Hara et al. [29] found one SNP (rs10252228) associated with regular leisure time physical activity. This SNP was located in the intergenic region between *NPSR1* and *DPY19L1*, and the SNP was also significant in replication samples. Heritability estimates varied from 1.3% in the study by Hara et al. [29] who used self-report to measure leisure time physical activity, to 21% for overall activity in the study by Doherty et al. [27] who used accelerometry (online supplementary 4).

Three of the GWAS also included candidate gene analysis of genes that previously have been reported to be associated with physical activity [28, 29, 31]. Lin et al. [31] reported a statistically significant association (*p* < 5 × 10^− 3^) for SNPs in several loci, including SNPs close to *GABRG3*, *CYP19A1*, *PAPSS2* and *CASR*. Hara et al. [29] found a weak association for a SNP in *DNAPTP6* with leisure time physical activity, but the association was not statistically significant after Bonferroni correction (*p* < 0.05/6). De Moor et al. [28] reported statistically significant associations (*p* < 0.01) for SNPs in *LEPR* and *CYP19A1*.

#### Candidate gene studies

The candidate gene studies showed associations (*p* < 0.05) between variants in 30 different genes and physical activity and/or sedentary behaviour (Table 3 and online supplementary 5). The high-quality study by Bruneau et al. [32] found an association between walking distance per week and an insertion/deletion polymorphism of a 287-bp Alu repeat sequence within the intron 16 in *ACE* (rs4340). This polymorphism was also found to be associated with both physical activity and sedentary behaviour in two medium quality studies [43, 52] and in one low quality study [51]. However, the GWAS by Lin et al. [31] and De Moor et al. [28] did not successfully replicate SNPs in or close to the *ACE* gene. In another high quality candidate gene study, Bruneau et al. [33] found an association between light intensity physical activity and a SNP in *IL15RA* (rs2228059). In total, variants in nine candidate genes (*ACE, CASR, CYP19A, FTO, DRD2, CNR1, LEPR, MC4R, NPC1*) were found to be associated with physical activity or sedentary behaviour in more than one study. Variants in or close to *MC4R* was associated with physical activity in three medium quality studies [34, 40, 42]; however, the GWAS by De Moor et al. [28] and Lin et al. [31] did not report an association between physical activity and SNPs in the vicinity of the *MC4R* gene.

Online supplementary 6 shows effect size, standard error and *p*-value for genetic variants from candidate genes studies associated with accelerometry defined phenotypes reported in GWAS summary statistics [26, 27]. All *p*-values were above the conventional threshold of 5 × 10^− 8^; however, we observed that the *FTO* gene (rs9939609) reported to be associated with sitting time by Klimentidis et al. [39] and the *NPC1* (rs1805081) reported to be associated with physical activity level by Reddon et al. [47] had *p*-values of 0.04 and 0.002 for sedentary behaviour and overall activity, respectively.

## Discussion

This systematic review provides an overview of genetic variants associated with physical activity or sedentary behaviour. Fifty-four studies met the inclusion criteria, of which six studies were GWAS and 48 studies were candidate gene studies. While the quality scores for the GWAS were medium-to-high, most of the included candidate studies showed low-to-medium quality. The GWAS reported up to 10 loci that were significantly associated with physical activity or sedentary behaviour, and variants in nine candidate genes were found to be associated with physical activity or sedentary behaviour in more than one study. However, the available evidence was not consistent, and the included studies had several limitations that prevent us from drawing firm conclusions about valid and robust genotype-phenotype associations.

In line with previous reviews [12, 19, 20] we noted that phenotype definitions of physical activity varied considerable between studies, including constructs such as walking distance [32], low-intensity physical activity [45, 52], moderate intensity physical activity [34, 36, 49], vigorous physical activity [34, 37], energy expenditure [29, 31, 48], engagement in sports activities [44, 51], meeting recommended levels of physical activity [50], and physical activity level from childhood to adolescence [46]. Moreover, these phenotype definitions were in many studies based on instruments with poor validity. Likewise, phenotype definitions of sedentary behaviour were in several studies based on self-reports, which is shown to have very poor validity [53–55].

Self-reported measures of physical activity and sedentary behaviour are prone to measurement error and misclassifications [56] and findings on genotype-phenotype associations should therefore be interpreted with caution. Likewise, there are some limitations related to the use of objective measurements to define phenotypes that should be considered when interpreting the results [57–59]. For example, a single accelerometer may not capture all relevant activity [60, 61] and the use of different cut-offs points and methods for processing the accelerometer data are known to create large and significant differences in the estimated physical activity level [62, 63]. Thus, it is possible that the studies included in this review capture different aspects of physical activity and sedentary behaviour. Accordingly, the inconsistent findings across studies between genetic variants and physical activity or sedentary behaviour can partly be related to discrepancies in the measurements of physical activity and sedentary behaviour and the resulting phenotype definition.

Although recent advancements in methods and technology allow fast and accurate analyses of whole-genome samples [64, 65], only six GWAS have investigated genetic variants associated with physical activity. Moreover, only one GWAS has investigated genetics variants associates with sedentary behaviour and only two GWAS used objective measurements to define phenotypes. Several SNPs were associated with physical activity, but few SNPs or genes have been identified in more than one study. One exception is SNPs close to the *DPY19L1* gene, which was identified by two medium quality GWAS [29, 30]. The molecular mechanism behind the association between *DPY19L1* and physical behaviour remains elusive. *DPY19L1* may be required for a proper radial migration of glutamatergic neuron, a major excitatory component of the mammalian neocortex [66]. Hara et al. [29] found an association between self-reported physical activity and the rs10252228 SNP, which is located in the intergenic region between *NPSR1* and *DPY19L1.* This study comprised individuals of Japanese ancestry and the findings were confirmed in replication samples. Likewise, Klimentidis et al. [30] found an association between the rs328902 SNP close to the *DPY19L1* and self-reported leisure time physical activity. However, the genetic effect sizes in the latter study were small, and the replication cohort was considered insufficiently powered to replicate the associations. It should also be noted that the two GWAS by Klimentidis et al. [30] and Doherty et al. [27] are based on the same data from UK Biobank. The few GWAS performed to date, along with the variable study size, different phenotypes of physical behaviours, and the wide range of ethnicities (e.g., Caucasians, Japanese, and African American) makes it difficult to compare the GWAS. Moreover, a GWAS require a large sample size to be adequately powered to adopt a significance level that account for multiple testing [15]. With a recommended genome-wide significance threshold of *p* < 5 × 10^− 8^ [67, 68], most GWAS in this review were underpowered to detect all the possible heritability explained by the SNPs (three out of six GWAS used the recommended threshold level of *p* < 5 × 10^− 8^). Moreover, GWAS have been criticized because markers across genomes with no direct biological relevance to the phenotype of interest could be located [15]. Nevertheless, this is a rapid growing area of research and one can overcome several limitations by larger sample sizes and advancements in technology, methodology and computing. Future studies may therefore have the potential to identify missing signals, account for population stratification, identify rare mutations, identify gene-environment interactions, and correspondingly, explain more of the heritability [15, 65].

Despite the widespread use of candidate gene studies, our review shows that this approach has produced only a few replicated associations related to physical activity or sedentary behaviour. Nine out of 30 candidate genes were found to be associated with physical activity in more than one study. The explanation for these inconsistent findings may be linked to the small study samples and the heterogeneity of the definitions of physical activity phenotypes. Population-based candidate gene studies with large study samples with adequate statistical power were rare. Most candidate gene studies had rather small sample sizes, and the likelihood of identifying a true genetic variant may therefore be low.

There may exist a complex set of genetic, environmental, and phenotypic factors that connect physical activity and sedentariness to other behavioural traits [40, 69, 70], and we cannot exclude the possibility of pleiotropic effects (i.e., a single genetic variant affecting multiple traits), nor that these effects are influenced by the phenotype definitions. For instance, two candidate gene studies reported an association between FTO and self-reported physical activity and time spent sitting [35, 39]. However, these findings were not supported by studies using objective measurements [41] or a more well-defined physical activity index [38]. It has been argued that candidate gene studies are insufficient for identifying the genetic contribution to variation in physical activity [71] and that the genetic susceptibility to a physically active or inactive lifestyle should be studied in the context of social and environmental factors [11, 19, 72], i.e., gene-environment interactions are expected to explain some of the unexplained heritability [73, 74]. In most of the included studies, the conclusions were based on *p*-values and many studies did not present an estimate for the associations under study, making it impossible to make a judgement about the strength of the association. Together with predominantly small sample sizes this might introduce biased results. It is also possible that the strong focus on *p*-values leads to publication bias and selective reporting [21]. The findings from the candidate gene studies should therefore be interpreted in view of unclear or unknown effect sizes, small study samples, and the possible influence of sociodemographic and environmental factors.

Strengths of the current systematic review include the comprehensive literature search in two bibliographic databases supervised by a trained research librarian, the use of checklists to assess risk of bias/methodological quality and blinding of reviewers during data extraction. However, the quality assessment could be problematic since all potential sources for bias are weighted equally. Since bias in genetic association studies are not completely understood, evidence of what study characteristics that are most important is lacking [21]. Another limitation is that we only retrieved associations from candidate gene studies that were nominally statistically significant (i.e., *p*-value < 0.05), since most authors only showed statistically significant results. Thus, potentially important associations from small studies may have been omitted from this review. Furthermore, although we excluded studies that only reported physical activity related to active transport or occupational activity, few studies reported whether occupational physical activity or work-related sedentariness were included in their measurements. This might bias the reported associations since occupational physical activity can be constrained by the type of occupation and work tasks. Obtaining accurate and detailed measurements of physical activity behaviour (type of activity, duration, intensity, frequency, and domains [leisure, work, transportation]) are critical to understand the genetic contribution to physical activity behaviour. This is underlined by heritability being greater in studies using objective measurements of physical behaviour [27, 29, 30]. Future studies should therefore aim at using objective measurements to obtain more well-defined phenotypes, enabling identification of more robust genetic instruments for physical activity behaviour. This could in turn provide the basis for Mendelian randomisation studies to improve causal inference about the effect of physical activity and sedentary behaviour on morbidity and mortality, and thus evade some of the central challenges of conventional epidemiological studies, such as confounding, reverse causation and measurement error [16].

This systematic review shows that several genetic variants are associated with physical activity or sedentary behaviour. However, findings across studies are inconsistent and the results should be interpreted with caution due to methodological shortcomings, such as the large variation in phenotype definitions, study designs, and study populations. Moreover, replications issues are prominent in this field and there is general lack of high-quality studies. Thus, our review highlights the need for more high-quality GWAS with consistent phenotype definitions using objective measurements to elucidate the genetic influence on physical activity and sedentary behaviour.

## Supplementary Information

**Additional file 1.** Search strategies.

**Additional file 2.** Data extraction form.

**Additional file 3.** References for included articles.

**Additional file 4.** Overview of genotype-phenotype associations for all included GWAS.

**Additional file 5.** Overview of associations between candidate genes and physical activity or sedentary behavior in all included candidate gene studies.

**Additional file 6. **Effect size, standard error and p-value for genetic variants from candidate genes studies associated with accelerometry defined phenotypes reported in summary statistics from the high quality GWAS.

## Data Availability

Not applicable.

## Abbreviations

DNA: Deoxyribonucleic acid
GWAS: Genome wide association study
PRISMA: Preferred reporting items for systematic reviews and meta-analyses
SNP: Single nucleotide polymorphisms

## References

[1] Kohl HW, Craig CL, Lambert EV, Inoue S, Alkandari JR, Leetongin G (2012). The pandemic of physical inactivity: global action for public health. Lancet.

[2] Lee IM, Shiroma EJ, Lobelo F, Puska P, Blair SN, Katzmarzyk PT (2012). Effect of physical inactivity on major non-communicable diseases worldwide: an analysis of burden of disease and life expectancy. Lancet.

[3] WHO (2012). Political declaration of the high-level meeting of the general assembly on the prevention and control of non-communicable diseases (resolution 66/2).

[4] Strain T, Brage S, Sharp SJ, Richards J, Tainio M, Ding D (2020). Use of the prevented fraction for the population to determine deaths averted by existing prevalence of physical activity: a descriptive study. Lancet Glob Health.

[5] Arem H, Moore SC, Patel A, Hartge P, Berrington de Gonzalez A, Visvanathan K (2015). Leisure time physical activity and mortality: a detailed pooled analysis of the dose-response relationship. JAMA Intern Med.

[6] Zhang D, Liu X, Liu Y, Sun X, Wang B, Ren Y (2017). Leisure-time physical activity and incident metabolic syndrome: a systematic review and dose-response meta-analysis of cohort studies. Metabolism.

[7] Moore SC, Lee IM, Weiderpass E, Campbell PT, Sampson JN, Kitahara CM (2016). Association of Leisure-Time Physical Activity with Risk of 26 types of Cancer in 1.44 million adults. JAMA Intern Med.

[8] 2018 Physical Activity Guidelines Advisory Committee (2018). 2018 physical activity guidelines advisory committee scientific report.

[9] Kraus WE, Powell KE, Haskell WL, Janz KF, Campbell WW, Jakicic JM (2019). Physical activity, all-cause and cardiovascular mortality, and cardiovascular disease. Med Sci Sports Exerc.

[10] Troiano RP, Berrigan D, Dodd KW, Masse LC, Tilert T, McDowell M (2008). Physical activity in the United States measured by accelerometer. Med Sci Sports Exerc.

[11] Lightfoot JT, EJC DEG, Booth FW, Bray MS, Kaprio J (2018). Biological/genetic regulation of physical activity level: consensus from GenBioPAC. Med Sci Sports Exerc.

[12] Santos DM, Katzmarzyk PT, Seabra AF, Maia JA (2012). Genetics of physical activity and physical inactivity in humans. Behav Genet.

[13] Silfee VJ, Haughton CF, Jake-Schoffman DE, Lopez-Cepero A, May CN, Sreedhara M (2018). Objective measurement of physical activity outcomes in lifestyle interventions among adults: a systematic review. Prev Med Rep.

[14] Dowd KP, Szeklicki R, Minetto MA, Murphy MH, Polito A, Ghigo E (2018). A systematic literature review of reviews on techniques for physical activity measurement in adults: a DEDIPAC study. Int J Behav Nutr Phys Act.

[15] Tam V, Patel N, Turcotte M, Bossé Y, Paré G, Meyre D (2019). Benefits and limitations of genome-wide association studies. Nat Rev Genet.

[16] Davies NM, Holmes MV, Davey SG (2018). Reading Mendelian randomisation studies: a guide, glossary, and checklist for clinicians. BMJ.

[17] Wade KH, Richmond RC, Davey SG (2018). Physical activity and longevity: how to move closer to causal inference. Br J Sports Med.

[18] Herring MP, Sailors MH, Bray MS (2014). Genetic factors in exercise adoption, adherence and obesity. Obes Rev.

[19] Lin X, Eaton CB, Manson JE, Liu S (2017). The genetics of physical activity. Curr Cardiol Rep.

[20] Zhang X, Speakman JR (2019). Genetic factors associated with human physical activity: are your genes too tight to prevent you exercising?. Endocrinology.

[21] Sagoo GS, Little J, Higgins JP (2009). Systematic reviews of genetic association studies. Human Genome Epidemiology Network. PLoS Med.

[22] Liberati A, Altman DG, Tetzlaff J, Mulrow C, Gotzsche PC, Ioannidis JP (2009). The PRISMA statement for reporting systematic reviews and meta-analyses of studies that evaluate healthcare interventions: explanation and elaboration. BMJ.

[23] Bramer WM, Milic J, Mast F (2017). Reviewing retrieved references for inclusion in systematic reviews using EndNote. J Med Libr Assoc.

[24] Eskola PJ, Lemmela S, Kjaer P, Solovieva S, Mannikko M, Tommerup N (2012). Genetic association studies in lumbar disc degeneration: a systematic review. PLoS One.

[25] Hayden JA, van der Windt DA, Cartwright JL, Cote P, Bombardier C (2013). Assessing bias in studies of prognostic factors. Ann Intern Med.

[26] Doherty A. Summary statistics relating to “GWAS identifies 14 loci for device-measured physical activity and sleep duration”. Oxford: University of Oxford; 2018. 10.1038/s41467-018-07743-4 PMC6288145 30531941

[27] Doherty A, Smith-Byrne K, Ferreira T, Holmes MV, Holmes C, Pulit SL (2018). GWAS identifies 14 loci for device-measured physical activity and sleep duration. Nat Commun.

[28] De Moor MH, Liu YJ, Boomsma DI, Li J, Hamilton JJ, Hottenga JJ (2009). Genome-wide association study of exercise behavior in Dutch and American adults. Med Sci Sports Exerc.

[29] Hara M, Hachiya T, Sutoh Y, Matsuo K, Nishida Y, Shimanoe C (2018). Genomewide association study of leisure-time exercise behavior in Japanese adults. Med Sci Sports Exerc.

[30] Klimentidis YC, Raichlen DA, Bea J, Garcia DO, Wineinger NE, Mandarino LJ (2018). Genome-wide association study of habitual physical activity in over 377,000 UK biobank participants identifies multiple variants including CADM2 and APOE. Int J Obes.

[31] Lin X, Chan KK, Huang YT, Luo XI, Liang L, Wilson J (2018). Genetic determinants for leisure-time physical activity. Med Sci Sports Exerc.

[32] Bruneau M, Angelopoulos TJ, Gordon P, Moyna N, Visich P, Zoeller R (2017). The angiotensin-converting enzyme insertion/deletion polymorphism rs4340 associates with habitual physical activity among European American adults. Mol Genet Genomic Med.

[33] Bruneau M, Walsh S, Selinsky E, Ash G, Angelopoulos TJ, Clarkson P (2018). A genetic variant in IL-15Ralpha correlates with physical activity among European-American adults. Mol Genet Genomic Med.

[34] Cole SA, Butte NF, Voruganti VS, Cai G, Haack K, Kent JW (2010). Evidence that multiple genetic variants of MC4R play a functional role in the regulation of energy expenditure and appetite in Hispanic children. Am J Clin Nutr.

[35] Camps S, Verhoef SPM, Bouwman FG, Mariman ECM, Westerterp KR (2019). Association of FTO and ADRB2 gene variation with energy restriction induced adaptations in resting energy expenditure and physical activity. Gene X.

[36] Flack K, Pankey C, Ufholz K, Johnson L, Roemmich JN (2019). Genetic variations in the dopamine reward system influence exercise reinforcement and tolerance for exercise intensity. Behav Brain Res.

[37] Gielen M, Westerterp-Plantenga MS, Bouwman FG, Joosen AM, Vlietinck R, Derom C (2014). Heritability and genetic etiology of habitual physical activity: a twin study with objective measures. Genes Nutr.

[38] Hakanen M, Raitakari OT, Lehtimaki T, Peltonen N, Pahkala K, Sillanmaki L (2009). FTO genotype is associated with body mass index after the age of seven years but not with energy intake or leisure-time physical activity. J Clin Endocrinol Metab.

[39] Klimentidis YC, Arora A, Chougule A, Zhou J, Raichlen DA (2016). FTO association and interaction with time spent sitting. Int J Obes.

[40] Lee H, Ash GI, Angelopoulos TJ, Gordon PM, Moyna NM, Visich PS (2015). Obesity-related genetic variants and their associations with physical activity. Sports Med Open.

[41] Liu G, Zhu H, Lagou V, Gutin B, Stallmann-Jorgensen IS, Treiber FA (2010). FTO variant rs9939609 is associated with body mass index and waist circumference, but not with energy intake or physical activity in European- and African-American youth. BMC Med Genet.

[42] Loos RJ, Rankinen T, Tremblay A, Perusse L, Chagnon Y, Bouchard C (2005). Melanocortin-4 receptor gene and physical activity in the Quebec family study. Int J Obes.

[43] Maestu J, Latt E, Raask T, Sak K, Laas K, Jurimae J (2013). Ace I/D polymorphism is associated with habitual physical activity in pubertal boys. J Physiol Sci.

[44] Many GM, Kendrick Z, Deschamps CL, Sprouse C, Tosi LL, Devaney JM (2017). Genetic characterization of physical activity behaviours in university students enrolled in kinesiology degree programs. Appl Physiol Nutr Metab.

[45] Murakami H, Iemitsu M, Fuku N, Sanada K, Gando Y, Kawakami R (2014). The Q223R polymorphism in the leptin receptor associates with objectively measured light physical activity in free-living Japanese. Physiol Behav.

[46] Murakami H, Fuku N, Kawakami R, Gando Y, Iemitsu M, Sanada K (2017). DRD2/ANKK1 gene polymorphism rs1800497 is associated with exercise habit in the period from childhood to adolescence in Japanese. J Sports Med Phys Fitness.

[47] Reddon H, Gerstein HC, Engert JC, Mohan V, Bosch J, Desai D (2016). Physical activity and genetic predisposition to obesity in a multiethnic longitudinal study. Sci Rep.

[48] Richert L, Chevalley T, Manen D, Bonjour JP, Rizzoli R, Ferrari S (2007). Bone mass in prepubertal boys is associated with a Gln223Arg amino acid substitution in the leptin receptor. J Clin Endocrinol Metab.

[49] Van Deveire KN, Scranton SK, Kostek MA, Angelopoulos TJ, Clarkson PM, Gordon PM (2012). Variants of the ankyrin repeat domain 6 gene (ANKRD6) and muscle and physical activity phenotypes among European-derived American adults. J Strength Cond Res.

[50] Wilkinson AV, Gabriel KP, Wang J, Bondy ML, Dong Q, Wu X (2013). Sensation-seeking genes and physical activity in youth. Genes Brain Behav.

[51] Winnicki M, Accurso V, Hoffmann M, Pawlowski R, Dorigatti F, Santonastaso M (2004). Physical activity and angiotensin-converting enzyme gene polymorphism in mild hypertensives. Am J Med Genet A.

[52] Wong WP, Zhao Y, Koh WP (2012). Gene polymorphism in angiotensin-I-converting enzyme and physical activity among normotensive Chinese. Int J Sport Nutr Exerc Metab.

[53] Lagersted-Olsen J, Korshoj M, Skotte J, Carneiro IG, Sogaard K, Holtermann A (2014). Comparison of objectively measured and self-reported time spent sitting. Int J Sports Med.

[54] Gupta N, Christiansen CS, Hanisch C, Bay H, Burr H, Holtermann A (2017). Is questionnaire-based sitting time inaccurate and can it be improved? A cross-sectional investigation using accelerometer-based sitting time. BMJ Open.

[55] Pedersen SJ, Kitic CM, Bird ML, Mainsbridge CP, Cooley PD (2016). Is self-reporting workplace activity worthwhile? Validity and reliability of occupational sitting and physical activity questionnaire in desk-based workers. BMC Public Health.

[56] Helmerhorst HJ, Brage S, Warren J, Besson H, Ekelund U (2012). A systematic review of reliability and objective criterion-related validity of physical activity questionnaires. Int J Behav Nutr Phys Act.

[57] Strath SJ, Kaminsky LA, Ainsworth BE, Ekelund U, Freedson PS, Gary RA (2013). Guide to the assessment of physical activity: clinical and research applications: a scientific statement from the American Heart Association. Circulation.

[58] Baumann S, Groß S, Voigt L, Ullrich A, Weymar F, Schwaneberg T (2018). Pitfalls in accelerometer-based measurement of physical activity: the presence of reactivity in an adult population. Scand J Med Sci Sports.

[59] Pedišić Ž, Bauman A (2015). Accelerometer-based measures in physical activity surveillance: current practices and issues. Br J Sports Med.

[60] Matthews CE, Hagströmer M, Pober DM, Bowles HR (2012). Best practices for using physical activity monitors in population-based research. Med Sci Sports Exerc.

[61] Cleland I, Kikhia B, Nugent C, Boytsov A, Hallberg J, Synnes K (2013). Optimal placement of accelerometers for the detection of everyday activities. Sensors.

[62] Orme M, Wijndaele K, Sharp SJ, Westgate K, Ekelund U, Brage S (2014). Combined influence of epoch length, cut-point and bout duration on accelerometry-derived physical activity. Int J Behav Nutr Phys Act.

[63] Leeger-Aschmann CS, Schmutz EA, Zysset AE, Kakebeeke TH, Messerli-Bürgy N, Stülb K (2019). Accelerometer-derived physical activity estimation in preschoolers - comparison of cut-point sets incorporating the vector magnitude vs the vertical axis. BMC Public Health.

[64] Visscher PM, Brown MA, McCarthy MI, Yang J (2012). Five years of GWAS discovery. Am J Hum Genet.

[65] Visscher PM, Wray NR, Zhang Q, Sklar P, McCarthy MI, Brown MA (2017). 10 years of GWAS discovery: biology, function, and translation. Am J Hum Genet.

[66] Watanabe K, Takebayashi H, Bepari AK, Esumi S, Yanagawa Y, Tamamaki N (2011). Dpy19l1, a multi-transmembrane protein, regulates the radial migration of glutamatergic neurons in the developing cerebral cortex. Development.

[67] Dudbridge F, Gusnanto A (2008). Estimation of significance thresholds for genomewide association scans. Genet Epidemiol.

[68] Manolio TA, Collins FS, Cox NJ, Goldstein DB, Hindorff LA, Hunter DJ (2009). Finding the missing heritability of complex diseases. Nature.

[69] Thomas D (2010). Gene--environment-wide association studies: emerging approaches. Nat Rev Genet.

[70] Bookman EB, McAllister K, Gillanders E, Wanke K, Balshaw D, Rutter J (2011). Gene-environment interplay in common complex diseases: forging an integrative model—recommendations from an NIH workshop. Genet Epidemiol.

[71] Bouchard C (2011). Overcoming barriers to progress in exercise genomics. Exerc Sport Sci Rev.

[72] Pérusse L, Tremblay A, Leblanc C, Bouchard C (1989). Genetic and environmental influences on level of habitual physical activity and exercise participation. Am J Epidemiol.

[73] Aschard H, Hancock DB, London SJ, Kraft P (2010). Genome-wide meta-analysis of joint tests for genetic and gene-environment interaction effects. Hum Hered.

[74] Murcray CE, Lewinger JP, Gauderman WJ. Gene-environment interaction in genome-wide association studies. Am J Epidemiol. 2009;169(2):219–26. 10.1093/aje/kwn353 PMC2732981 19022827

[75] Kim J, Kim J, Min H, Oh S, Kim Y, Lee AH, et al. Joint identification of genetic variants for physical activity in Korean population. Intern J Mol Sci. 2014;15(7):12407–21. 10.3390/ijms150712407 PMC4139850 25026172

[76] Adamska-Patruno E, Goscik J, Czajkowski P, Maliszewska K, Ciborowski M, Golonko A, et al. The MC4R genetic variants are associated with lower visceral fat accumulation and higher postprandial relative increase in carbohydrate utilization in humans. Eur J Nutr. 2019;58(7):2929–41. 10.1007/s00394-019-01955-0 PMC6768895 30945034

[77] Berentzen T, Kring SII, Holst C, Zimmermann E, Jess T, Hansen T, et al. Lack of association of fatness-related FTO gene variants with energy expenditure or physical activity. Journal Clin Endocrinol Metab. 2008;93(7):2904–8. 10.1210/jc.2008-0007 18445669

[78] Boer JM, Ehnholm C, Menzel HJ, Havekes LM, Rosseneu M, O'Reilly DS, et al. Interactions between lifestyle-related factors and the ApoE polymorphism on plasma lipids and apolipoproteins. The EARS Study. European Atherosclerosis Research Study. Arterioscler Thromb Vasc Biol. 1997;17(9):1675–81. 10.1161/01.atv.17.9.1675 9327762

[79] Espinosa-Salinas I, de la Iglesia R, Colmenarejo G, Molina S, Reglero G, Martinez JA, et al. GCKR rs780094 polymorphism as a genetic variant involved in physical exercise. Genes. 2019;10(8). 10.3390/genes10080570 PMC6722860 31357711

[80] Fonseca-Portilla R, Krell-Roesch J, Shaibi GQ, Caselli RJ, Mandarino LJ, Zhang N, et al. Brain-Derived Neurotrophic Factor and Its Associations with Metabolism and Physical Activity in a Latino Sample. Metab Syndr Relat Disord. 2019;17(2):75–80. 10.1089/met.2018.0028 PMC6484340 30418087

[81] Fuentes RM, Perola M, Nissinen A, Tuomilehto J. ACE gene and physical activity, blood pressure, and hypertension: a population study in Finland. J Appl Physiol (1985). 2002;92(6):2508–12. 10.1152/japplphysiol.01196.2001 12015366

[82] Goleva-Fjellet S, Bjurholt AM, Kure EH, Larsen IK, Storen O, Saebo M. Distribution of allele frequencies for genes associated with physical activity and/or physical capacity in a homogenous Norwegian cohort- a cross-sectional study. BMC Genetics. 2020;21(1). 10.1186/s12863-020-0813-1 PMC6979285 31973699

[83] Good DJ, Li M, Deater-Deckard K. A Genetic Basis for Motivated Exercise. Exerc Sport Sci Rev. 2015;43(4):231–7. 10.1249/JES.0000000000000057 26196864

[84] Grady DL, Thanos PK, Corrada MM, Barnett JC, Jr., Ciobanu V, Shustarovich D, et al. DRD4 genotype predicts longevity in mouse and human. J Neurosci. 2013;33(1):286-91. 10.1523/JNEUROSCI.3515-12.2013 PMC3710129 23283341

[85] Haber E, Słowińska-Lisowska M, Jóźkow P, Łaczmański Ł, Mędraś M. Relationships Between the G861C Polymorphism of the 5-HT1B Serotonin Receptor Gene and the Physical Activity in Men. Adv Clin Exp Med. 2010;19(4):455-9.

[86] Harbron J, van der Merwe L, Zaahl MG, Kotze MJ, Senekal M. Fat mass and obesity-associated (FTO) gene polymorphisms are associated with physical activity, food intake, eating behaviors, psychological health, and modeled change in body mass index in overweight/obese Caucasian adults. Nutrients. 2014;6(8):3130–52. 10.3390/nu6083130 PMC4145299 25102252

[87] Hubacek JA, Pikhart H, Peasey A, Kubinova R, Bobak M. FTO variant, energy intake, physical activity and basal metabolic rate in Caucasians. The HAPIEE study. Physiol Res 2011;60(1):175–83. 10.33549/physiolres.932066 PMC3955871 20945952

[88] Huppertz C, Bartels M, Groen-Blokhuis MM, Dolan CV, de Moor MH, Abdellaoui A, et al. The dopaminergic reward system and leisure time exercise behavior: a candidate allele study. BioMed research international. 2014;2014:591717. 10.1155/2014/591717 PMC3964758 24734235

[89] Jensen HA, Harslof LB, Nielsen MS, Christensen LB, Ritz C, Michaelsen KF, et al. FADS single-nucleotide polymorphisms are associated with behavioral outcomes in children, and the effect varies between sexes and is dependent on PPAR genotype. Am J Clin Nutr. 2014;100(3):826–32. 10.3945/ajcn.114.087882 25080457

[90] Jozkow P, Slowinska-Lisowska M, Laczmanski L, Medras M, Trzmiel A, Kuliczkowska-Plaksej J. CAG repeat polymorphism in the androgen receptor gene and the level of physical activity (HALS Study). The Journal of sports medicine and physical fitness. 2009;49(4):453–8. 20087306

[91] Jozkow P, Slowinska-Lisowska M, Laczmanski L, Jakubiec D, Medras M. Melanocortin-4 receptor gene polymorphism and the level of physical activity in men (HALS Study). Endocrine. 2011;39(1):62–8. 10.1007/s12020-010-9412-7 21046283

[92] Jozkow P, Slowinska-Lisowska M, Laczmanski L, Medras M. DRD2 C313T and DRD4 48-bp VNTR polymorphisms and physical activity of healthy men in Lower Silesia, Poland (HALS study). Annals of human biology. 2013;40(2):186–90. 10.3109/03014460.2012.748829 23215759

[93] Kirac D, Kasimay Cakir O, Avcilar T, Deyneli O, Kurtel H, Yazici D, et al. Effects of MC4R, FTO, and NMB gene variants to obesity, physical activity, and eating behavior phenotypes. IUBMB life. 2016;68(10):806–16. 10.1002/iub.1558 27634552

[94] Lorentzon M, Lorentzon R, Lerner UH, Nordstrom P. Calcium sensing receptor gene polymorphism, circulating calcium concentrations and bone mineral density in healthy adolescent girls. Eur J Endocrinol. 2001;144(3):257–61. 10.1530/eje.0.1440257 11248745

[95] Luglio HF, Eurike D, Huriyati E, Julia M, Susilowati R. Gene-lifestyle interaction: The role of SNPs in UCP2-866G/A and UCP3-55C/T on dietary intake and physical activity in Indonesian obese female adolescents. Mediterranean Journal of Nutrition and Metabolism. 2016;9(2):87–93.

[96] Moleres A, Rendo-Urteaga T, Azcona C, Martinez JA, Gomez-Martinez S, Ruíz JR, et al. Il6 gene promoter polymorphism (-174G/C) influences the association between fat mass and cardiovascular risk factors. J Physiol Biochem. 2009;65(4):405–13. 10.1007/BF03185936 20358354

[97] Salmen T, Heikkinen AM, Mahonen A, Kroger H, Komulainen M, Pallonen H, et al. Relation of aromatase gene polymorphism and hormone replacement therapy to serum estradiol levels, bone mineral density, and fracture risk in early postmenopausal women. Ann Med. 2003;35(4):282–8. 10.1080/07853890310006370 12846271

[98] Simonen RL, Rankinen T, Perusse L, Leon AS, Skinner JS, Wilmore JH, et al. A dopamine D2 receptor gene polymorphism and physical activity in two family studies. Physiol Behav. 2003;78(4-5):751–7. 10.1016/s0031-9384(03)00084-2 12782232

[99] Van der Mee DJ, Fedko IO, Hottenga J-J, Ehli EA, Van Der Zee MD, Ligthart L, et al. Dopaminergic genetic variants and voluntary externally paced exercise behavior. Med Sci Sports Exerc. 2018;50(4):700. 10.1249/MSS.0000000000001479 PMC5856580 29135816

[100] Vimaleswaran KS, Franks PW, Brage S, Grontved A, Wareham NJ, Ekelund U, et al. Lack of association between PCK1 polymorphisms and obesity, physical activity, and fitness in European Youth Heart Study (EYHS). Obesity (Silver Spring, Md). 2010;18(10):1975–80. 10.1038/oby.2010.13 20134411

[101] Walsh S, Haddad CJ, Kostek MA, Angelopoulos TJ, Clarkson PM, Gordon PM, et al. Leptin and leptin receptor genetic variants associate with habitual physical activity and the arm body composition response to resistance training. Gene. 2012;510(1):66–70. 10.1016/j.gene.2012.08.020 PMC3500611 22975643

[102] West NR, Dorling J, Thackray AE, Hanson NC, Decombel SE, Stensel DJ, et al. Effect of Obesity-Linked FTO rs9939609 Variant on Physical Activity and Dietary Patterns in Physically Active Men and Women. J Obes. 2018;2018:7560707. 10.1155/2018/7560707 PMC5852866 29686893

