# Supplementary material for: Genetic variants related to physical activity or sedentary behaviour: a systematic review
Source: Int J Behav Nutr Phys Act. 2021 Jan 22;18:15. doi: 10.1186/s12966-020-01077-5 (PMC7821484; doi:10.1186/s12966-020-01077-5)
Supplement: Supplementary file 1 — Additional file 1. Search strategies. [file 12966_2020_1077_MOESM1_ESM.docx]

**Online supplementary 1**

Search strategies

PubMed

(exercise [mh] OR exercis* [ti] OR exercis* [ot] OR "physical activity" [tiab] OR walk* [ti] OR walk* [ot] OR sedentary lifestyle [mh] OR sedentar* [tiab] OR sitting [tiab] OR "physical inactivity" [tiab]) AND (alleles [mh] OR genetic association studies [mh] OR genetic markers [mh] OR genetic pleiotropy [mh] OR polymorphism, genetic [mh] OR snp* [ti] OR snp* [ot] OR gwas [ti] OR gwas [ot] OR allele*[ti] OR allele*[ot] OR ((genetic[ti] OR genome[ti]) AND association[ti] AND (study[ti] OR studies[ti])) OR ((genetic[ot] OR genome[ot]) AND association[ot] AND (study[ot] OR studies[ot])) OR (genetic[ti] AND marker*[ti]) OR (genetic[ot] AND marker*[ot]) OR pleiotrop*[ti] OR pleiotrop*[ot] OR polymorphism*[ti] OR polymorphism*[ot]) AND ("1990/01/01"[PDAT] : "3000/12/31"[PDAT]) AND English [lang] NOT (review [pt] OR "case reports"[pt] OR editorial [pt] OR (animals[mh] NOT humans [mh]))

Embase

(exp exercise/ or exp physical activity/ or physical inactivity/ or sedentary lifestyle/ or (physical activity or sedentar* or sitting or physical inactivity).ti,kw,ab. or (exercis* or walk*).ti,kw.) and (exp allele/ or exp genetic association studies/ or exp genetic marker/ or exp genetic polymorphism/ or pleiotropy/ or (snp* or gwas or allele* or ((genetic or genome) *adj* association *adj* (study or studies)) or genetic marker* or pleiotrop* or polymorphism*).ti,kw.) and (english.la. and human/ and (199* or 20*).yr.) not (case study/ or case report/ or (editorial or review).pt.)
