# Supplementary material for: Genetic variants related to physical activity or sedentary behaviour: a systematic review
Source: Int J Behav Nutr Phys Act. 2021 Jan 22;18:15. doi: 10.1186/s12966-020-01077-5 (PMC7821484; doi:10.1186/s12966-020-01077-5)
Supplement: Supplementary file 2 — Additional file 2. Data extraction form. [file 12966_2020_1077_MOESM2_ESM.docx]

**Online supplementary 2** Data extraction form

| MS title |  | | | | | |
| --- | --- | --- | --- | --- | --- | --- |
| Author |  | | | | | |
| Year |  | |  | | | |
| Reviewer initals |  | |  | | | |
|  | | | | | | |
| **Descriptives** | | | | | | |
| Country/-ies of origin |  | | | | | |
| Ethnicity/-ies |  | | | | | |
| Study population (patient/general population) |  | | | | | |
| Design (GWAS, candidate gene, other) |  | | | | | |
|  | | | | | | |
| Age, years | group name | min | max | mean | sd | median |
| Overall |  |  |  |  |  |  |
| Group 1 |  |  |  |  |  |  |
| Group 2 |  |  |  |  |  |  |
| Group 3 |  |  |  |  |  |  |
| Group 4 |  |  |  |  |  |  |
|  | | | | | | |
|  |  | | Women | | Men | |
| No. of participants (%) | gr name | Total N | N | % | N | % |
| Overall |  |  |  |  |  |  |
| Group 1 |  |  |  |  |  |  |
| Group 2 |  |  |  |  |  |  |
|  | | | | | | |
| Physical activity instrument |  | | | | | |
|  |  | | | | | |
| Genotyping method |  | | | | | |

| **Risk of bias** |  | | |
| --- | --- | --- | --- |
| Inclusion/exclusion criteria | Is the inclusion/exclusion criteria specified? | | |
|  | *Described* |  | *1* |
|  | *With a reference* |  | *½* |
|  | *Not stated* |  | *0* |
| Population stratification | Is population stratification addressed? | | |
|  | *Accounted for in analyses(e.g. principal component)* |  | *1* |
|  | *Selection based on ethnic or geographical origin* |  | *½* |
|  | *Not stated* |  | *0* |
| Sample size/power | Is sample size/power calculations reported? | | |
|  | *Yes* |  | *1* |
|  | *No* |  | *0* |
| DNA sampling/storage | Is the sampling procedure described? | | |
|  | *Yes* |  | *1* |
|  | *No* |  | *0* |
| Genotyping | Is the genotyping method described? | | |
|  | *Yes* |  | *1* |
|  | *No* |  | *0* |
|  | Is quality control described? | | |
|  | *Yes* |  | *1* |
|  | *No* |  | *0* |
|  | Was the genotyping blinded for physical activity status? | | |
|  | *Yes* |  | *1* |
|  | *Not stated* |  | *0* |
| Hardy-Weinberg eq. | Is Hardy-Weinberg equilibrium considered? | | |
|  | *Yes* |  | *1* |
|  | *No* |  | *0* |
| Physical activity | Is the assessment procedure described (or in a reference)? | | |
|  | *Yes* |  | *1* |
|  | *No* |  | *0* |
|  | Is the instrument validated (in own study or with a reference)? | | |
|  | *Yes* |  | *1* |
|  | *No* |  | *0* |
|  | Was physical activity objectively measured? | | |
|  | *Yes* |  | *1* |
|  | *No* |  | *0* |
| Analysis and reporting | Is the strength of association reported? | | |
|  | *Yes* |  | *1* |
|  | *No* |  | *0* |
|  | *Not applicable (GWAS)* |  |  |
|  | Is the results replicated within the study? |  | |
|  | *Yes* |  | *1* |
|  | *No* |  | *0* |
|  | *Not applicable (not GWAS)* |  |  |
|  | | | |
| **SUM** | Total score |  | 12 |

| **Results** |  | | | |
| --- | --- | --- | --- | --- |
| PA phenotype |  | | | |
| Gene |  |  |  |  |
| Association |  |  |  |  |
| CI |  |  |  |  |
| P-value |  |  |  |  |
|  | | | | |
| PA phenotype |  | | | |
| Gene |  |  |  |  |
| Association |  |  |  |  |
| CI |  |  |  |  |
| P-value |  |  |  |  |
|  | | | | |
| PA phenotype |  | | | |
| Gene(s) |  |  |  |  |
| Association |  |  |  |  |
| CI |  |  |  |  |
| P-value |  |  |  |  |
|  | | | | |
| PA phenotype |  | | | |
| Gene(s) |  |  |  |  |
| Association |  |  |  |  |
| CI |  |  |  |  |
| P-value |  |  |  |  |

| **Possible references** |  | | |
| --- | --- | --- | --- |
| No |  |  | |
| Yes |  |  | |
|  | | | |
| If Yes, | First author | Year | Title words |
|  |  |  |  |
|  |  |  |  |
|  |  |  |  |
|  |  |  |  |
|  |  |  |  |
