# Supplementary material for: Genetic variants related to physical activity or sedentary behaviour: a systematic review
Source: Int J Behav Nutr Phys Act. 2021 Jan 22;18:15. doi: 10.1186/s12966-020-01077-5 (PMC7821484; doi:10.1186/s12966-020-01077-5)
Supplement: Supplementary file 3 — Additional file 3. References for included articles. [file 12966_2020_1077_MOESM3_ESM.docx]

**Online supplementary 3**

**References for included articles**

1. Adamska-Patruno E, Goscik J, Czajkowski P, Maliszewska K, Ciborowski M, Golonko A, et al. The MC4R genetic variants are associated with lower visceral fat accumulation and higher postprandial relative increase in carbohydrate utilization in humans. European Journal of Nutrition. 2019;58(7):2929-41.

2. Berentzen T, Kring SII, Holst C, Zimmermann E, Jess T, Hansen T, et al. Lack of association of fatness-related FTO gene variants with energy expenditure or physical activity. The Journal of Clinical Endocrinology & Metabolism. 2008;93(7):2904-8.

3. Boer JM, Ehnholm C, Menzel HJ, Havekes LM, Rosseneu M, O'Reilly DS, et al. Interactions between lifestyle-related factors and the ApoE polymorphism on plasma lipids and apolipoproteins. The EARS Study. European Atherosclerosis Research Study. Arterioscler Thromb Vasc Biol. 1997;17(9):1675-81.

4. Bruneau M, Jr., Angelopoulos TJ, Gordon P, Moyna N, Visich P, Zoeller R, et al. The angiotensin-converting enzyme insertion/deletion polymorphism rs4340 associates with habitual physical activity among European American adults. Mol Genet Genomic Med. 2017;5(5):524-30.

5. Bruneau M, Jr., Walsh S, Selinsky E, Ash G, Angelopoulos TJ, Clarkson P, et al. A genetic variant in IL-15Ralpha correlates with physical activity among European-American adults. Mol Genet Genomic Med. 2018;6(3):401-8.

6. Camps SGJA, Verhoef SPM, Bouwman FG, Mariman ECM, Westerterp KR. Association of FTO and ADRB2 gene variation with energy restriction induced adaptations in resting energy expenditure and physical activity. Gene: X. 2019;3 (no pagination).

7. Cole SA, Butte NF, Voruganti VS, Cai G, Haack K, Kent Jr JW, et al. Evidence that multiple genetic variants of MC4R play a functional role in the regulation of energy expenditure and appetite in Hispanic children. The American journal of clinical nutrition. 2010;91(1):191-9.

8. De Moor MH, Liu YJ, Boomsma DI, Li J, Hamilton JJ, Hottenga JJ, et al. Genome-wide association study of exercise behavior in Dutch and American adults. Medicine and science in sports and exercise. 2009;41(10):1887-95.

9. Doherty A, Smith-Byrne K, Ferreira T, Holmes MV, Holmes C, Pulit SL, et al. GWAS identifies 14 loci for device-measured physical activity and sleep duration. Nat Commun. 2018;9(1):5257.

10. Espinosa-Salinas I, de la Iglesia R, Colmenarejo G, Molina S, Reglero G, Martinez JA, et al. GCKR rs780094 polymorphism as a genetic variant involved in physical exercise. Genes. 2019;10(8).

11. Flack K, Pankey C, Ufholz K, Johnson L, Roemmich JN. Genetic variations in the dopamine reward system influence exercise reinforcement and tolerance for exercise intensity. Behavioural Brain Research. 2019;375 (no pagination).

12. Fonseca-Portilla R, Krell-Roesch J, Shaibi GQ, Caselli RJ, Mandarino LJ, Zhang N, et al. Brain-Derived Neurotrophic Factor and Its Associations with Metabolism and Physical Activity in a Latino Sample. Metab Syndr Relat Disord. 2019;17(2):75-80.

13. Fuentes RM, Perola M, Nissinen A, Tuomilehto J. ACE gene and physical activity, blood pressure, and hypertension: a population study in Finland. J Appl Physiol (1985). 2002;92(6):2508-12.

14. Gielen M, Westerterp-Plantenga MS, Bouwman FG, Joosen AM, Vlietinck R, Derom C, et al. Heritability and genetic etiology of habitual physical activity: a twin study with objective measures. Genes Nutr. 2014;9(4):415.

15. Goleva-Fjellet S, Bjurholt AM, Kure EH, Larsen IK, Storen O, Saebo M. Distribution of allele frequencies for genes associated with physical activity and/or physical capacity in a homogenous Norwegian cohort- a cross-sectional study. BMC Genetics. 2020;21(1).

16. Good DJ, Li M, Deater-Deckard K. A Genetic Basis for Motivated Exercise. Exerc Sport Sci Rev. 2015;43(4):231-7.

17. Grady DL, Thanos PK, Corrada MM, Barnett JC, Jr., Ciobanu V, Shustarovich D, et al. DRD4 genotype predicts longevity in mouse and human. J Neurosci. 2013;33(1):286-91.

18. Haber E, Słowińska-Lisowska M, Jóźkow P, Łaczmański Ł, Mędraś M. Relationships Between the G861C Polymorphism of the 5-HT1B Serotonin Receptor Gene and the Physical Activity in Men. ADVANCES IN CLINICAL AND EXPERIMENTAL MEDICINE. 2010;19(4):455-9.

19. Hakanen M, Raitakari OT, Lehtimaki T, Peltonen N, Pahkala K, Sillanmaki L, et al. FTO genotype is associated with body mass index after the age of seven years but not with energy intake or leisure-time physical activity. J Clin Endocrinol Metab. 2009;94(4):1281-7.

20. Hara M, Hachiya T, Sutoh Y, Matsuo K, Nishida Y, Shimanoe C, et al. Genomewide Association Study of Leisure-Time Exercise Behavior in Japanese Adults. Med Sci Sports Exerc. 2018;50(12):2433-41.

21. Harbron J, van der Merwe L, Zaahl MG, Kotze MJ, Senekal M. Fat mass and obesity-associated (FTO) gene polymorphisms are associated with physical activity, food intake, eating behaviors, psychological health, and modeled change in body mass index in overweight/obese Caucasian adults. Nutrients. 2014;6(8):3130-52.

22. Hubacek JA, Pikhart H, Peasey A, Kubinova R, Bobak M. FTO variant, energy intake, physical activity and basal metabolic rate in Caucasians. The HAPIEE study. Physiological research. 2011;60(1):175-83.

23. Huppertz C, Bartels M, Groen-Blokhuis MM, Dolan CV, de Moor MH, Abdellaoui A, et al. The dopaminergic reward system and leisure time exercise behavior: a candidate allele study. BioMed research international. 2014;2014:591717.

24. Jensen HA, Harslof LB, Nielsen MS, Christensen LB, Ritz C, Michaelsen KF, et al. FADS single-nucleotide polymorphisms are associated with behavioral outcomes in children, and the effect varies between sexes and is dependent on PPAR genotype. The American journal of clinical nutrition. 2014;100(3):826-32.

25. Jozkow P, Slowinska-Lisowska M, Laczmanski L, Medras M, Trzmiel A, Kuliczkowska-Plaksej J. CAG repeat polymorphism in the androgen receptor gene and the level of physical activity (HALS Study). The Journal of sports medicine and physical fitness. 2009;49(4):453-8.

26. Jozkow P, Slowinska-Lisowska M, Laczmanski L, Jakubiec D, Medras M. Melanocortin-4 receptor gene polymorphism and the level of physical activity in men (HALS Study). Endocrine. 2011;39(1):62-8.

27. Jozkow P, Slowinska-Lisowska M, Laczmanski L, Medras M. DRD2 C313T and DRD4 48-bp VNTR polymorphisms and physical activity of healthy men in Lower Silesia, Poland (HALS study). Annals of human biology. 2013;40(2):186-90.

28. Kim J, Kim J, Min H, Oh S, Kim Y, Lee AH, et al. Joint identification of genetic variants for physical activity in Korean population. International journal of molecular sciences. 2014;15(7):12407-21.

29. Kirac D, Kasimay Cakir O, Avcilar T, Deyneli O, Kurtel H, Yazici D, et al. Effects of MC4R, FTO, and NMB gene variants to obesity, physical activity, and eating behavior phenotypes. IUBMB life. 2016;68(10):806-16.

30. Klimentidis, Y. C., Arora, A., Chougule, A., Zhou, J., & Raichlen, D. A. FTO association and interaction with time spent sitting. International Journal of Obesity.2016; 40(3), 411-416.

31. Klimentidis YC, Raichlen DA, Bea J, Garcia DO, Wineinger NE, Mandarino LJ, et al. Genome-wide association study of habitual physical activity in over 377,000 UK Biobank participants identifies multiple variants including CADM2 and APOE. International journal of obesity. 2018; 42(6),1161-1176.

32. Lee H, Ash GI, Angelopoulos TJ, Gordon PM, Moyna NM, Visich PS, et al. Obesity-Related Genetic Variants and their Associations with Physical Activity. Sports Med Open. 2015;1(1):34.

33. Lin X, Chan KK, Huang YT, Luo XI, Liang L, Wilson J, et al. Genetic Determinants for Leisure-Time Physical Activity. Med Sci Sports Exerc. 2018;50(8):1620-8.

34. Liu G, Zhu H, Lagou V, Gutin B, Stallmann-Jorgensen IS, Treiber FA, et al. FTO variant rs9939609 is associated with body mass index and waist circumference, but not with energy intake or physical activity in European- and African-American youth. BMC medical genetics. 2010;11:57.

35. Loos RJ, Rankinen T, Tremblay A, Perusse L, Chagnon Y, Bouchard C. Melanocortin-4 receptor gene and physical activity in the Quebec Family Study. International journal of obesity (2005). 2005;29(4):420-8.

36. Lorentzon M, Lorentzon R, Lerner UH, Nordstrom P. Calcium sensing receptor gene polymorphism, circulating calcium concentrations and bone mineral density in healthy adolescent girls. Eur J Endocrinol. 2001;144(3):257-61.

37. Luglio HF, Eurike D, Huriyati E, Julia M, Susilowati R. Gene-lifestyle interaction: The role of SNPs in UCP2-866G/A and UCP3-55C/T on dietary intake and physical activity in Indonesian obese female adolescents. Mediterranean Journal of Nutrition and Metabolism. 2016;9(2):87-93.

38. Maestu J, Latt E, Raask T, Sak K, Laas K, Jurimae J, et al. Ace I/D polymorphism is associated with habitual physical activity in pubertal boys. The journal of physiological sciences : JPS. 2013;63(6):427-34.

39. Many GM, Kendrick Z, Deschamps CL, Sprouse C, Tosi LL, Devaney JM, et al. Genetic characterization of physical activity behaviours in university students enrolled in kinesiology degree programs. Appl Physiol Nutr Metab. 2017;42(3):278-84.

40. Moleres A, Rendo-Urteaga T, Azcona C, Martinez JA, Gomez-Martinez S, Ruíz JR, et al. Il6 gene promoter polymorphism (-174G/C) influences the association between fat mass and cardiovascular risk factors. J Physiol Biochem. 2009;65(4):405-13.

41. Murakami H, Iemitsu M, Fuku N, Sanada K, Gando Y, Kawakami R, et al. The Q223R polymorphism in the leptin receptor associates with objectively measured light physical activity in free-living Japanese. Physiol Behav. 2014;129:199-204.

42. Murakami H, Fuku N, Kawakami R, Gando Y, Iemitsu M, Sanada K, et al. DRD2/ANKK1 gene polymorphism rs1800497 is associated with exercise habit in the period from childhood to adolescence in Japanese. The Journal of Physical Fitness and Sports Medicine. 2017;6(2):95-102.

43. Reddon H, Gerstein HC, Engert JC, Mohan V, Bosch J, Desai D, et al. Physical activity and genetic predisposition to obesity in a multiethnic longitudinal study. Scientific reports. 2016;6:18672.

44. Richert L, Chevalley T, Manen D, Bonjour JP, Rizzoli R, Ferrari S. Bone mass in prepubertal boys is associated with a Gln223Arg amino acid substitution in the leptin receptor. J Clin Endocrinol Metab. 2007;92(11):4380-6.

45. Salmen T, Heikkinen AM, Mahonen A, Kroger H, Komulainen M, Pallonen H, et al. Relation of aromatase gene polymorphism and hormone replacement therapy to serum estradiol levels, bone mineral density, and fracture risk in early postmenopausal women. Ann Med. 2003;35(4):282-8.

46. Simonen RL, Rankinen T, Perusse L, Leon AS, Skinner JS, Wilmore JH, et al. A dopamine D2 receptor gene polymorphism and physical activity in two family studies. Physiology & behavior. 2003;78(4-5):751-7.

47. Van der Mee DJ, Fedko IO, Hottenga J-J, Ehli EA, Van Der Zee MD, Ligthart L, et al. Dopaminergic genetic variants and voluntary externally paced exercise behavior. Med Sci Sports Exerc. 2018;50(4):700.

48. Van Deveire KN, Scranton SK, Kostek MA, Angelopoulos TJ, Clarkson PM, Gordon PM, et al. Variants of the ankyrin repeat domain 6 gene (ANKRD6) and muscle and physical activity phenotypes among European-derived American adults. Journal of strength and conditioning research. 2012;26(7):1740-8.

49. Vimaleswaran KS, Franks PW, Brage S, Grontved A, Wareham NJ, Ekelund U, et al. Lack of association between PCK1 polymorphisms and obesity, physical activity, and fitness in European Youth Heart Study (EYHS). Obesity (Silver Spring, Md). 2010;18(10):1975-80.

50. Walsh S, Haddad CJ, Kostek MA, Angelopoulos TJ, Clarkson PM, Gordon PM, et al. Leptin and leptin receptor genetic variants associate with habitual physical activity and the arm body composition response to resistance training. Gene. 2012;510(1):66-70.

51. West NR, Dorling J, Thackray AE, Hanson NC, Decombel SE, Stensel DJ, et al. Effect of Obesity-Linked FTO rs9939609 Variant on Physical Activity and Dietary Patterns in Physically Active Men and Women. J Obes. 2018;2018:7560707.

52. Wilkinson AV, Gabriel KP, Wang J, Bondy ML, Dong Q, Wu X, et al. Sensation-seeking genes and physical activity in youth. Genes, brain, and behavior. 2013;12(2):181-8.

53. Winnicki M, Accurso V, Hoffmann M, Pawlowski R, Dorigatti F, Santonastaso M, et al. Physical activity and angiotensin-converting enzyme gene polymorphism in mild hypertensives. American journal of medical genetics Part A. 2004;125a(1):38-44.

54. Wong WP, Zhao Y, Koh WP. Gene polymorphism in angiotensin-I-converting enzyme and physical activity among normotensive Chinese. International journal of sport nutrition and exercise metabolism. 2012;22(3):192-8.
