# Supplementary material for: Genetic variants related to physical activity or sedentary behaviour: a systematic review
Source: Int J Behav Nutr Phys Act. 2021 Jan 22;18:15. doi: 10.1186/s12966-020-01077-5 (PMC7821484; doi:10.1186/s12966-020-01077-5)
Supplement: Supplementary file 4 — Additional file 4. Overview of genotype-phenotype associations for all included GWAS. [file 12966_2020_1077_MOESM4_ESM.docx]

**Online supplementary 4**

Genotype-phenotype associations in GWAS. Studies sorted in descending order according to quality score (high to low).

| **1^st^ author (year)** | **Phenotype** | **SNP** | **Closest gene** | **p-value SNP in discovery cohort** | **p-value in replication cohort** | **Overall p-value^a^** | **Replication of candidate SNPs/genes** | **Heritability** | **Quality score (0-12)** |
| --- | --- | --- | --- | --- | --- | --- | --- | --- | --- |
| Doherty (2018) | Overall activity  Sedentary time | rs564819152  rs2696625  rs59499656  rs26579  rs25981  rs1858242  rs34858520 | *SKIDA1*  *KANSL1-AS1*  *SYT4*  *MEF2C-AS2*  *EFNA5*  *LOC105377146*  *CALN1* | 4.20x10^-9^  3.20x10^-12^  1.90x10^-9^  2.60x10^-9^  3.00x10^-9^  3.10x10^-9^  4.20x10^-9^ | N/A | N/A | N/A | Ranging from 10% (moderate intensity activity) to 21% (overall activity) | 9 |
| Klimentidis (2018) | Moderate-to-vigorous physical activity  Vigorous physical activity  Strenuous sports or other exercises  Accelerometry- average acceleration (vector magnitude) | rs429358  rs7804463  rs2035562  rs2988004  rs1248860  rs13243553  rs3781411  rs328902  rs62253088  rs166840  rs159544  rs75930676  rs55657917 | *APOE*  *EXOC4*  *CADM2*  *PAX5*  *CADM2*  *EXOC4*  *CTBP2*  *DPY19L1*  *CADM2*  *AKAP10*  *CTC-436P18.1*  *SIPA1L1*  *CRHR1* | 7.3 x10^-11^  4.1 x10^-10^  1.0 x10^-09^  2.4 x10^-11^  5.3 x10^-15^  2.4 x10^-09^  1.0 x10^-10^  1.3 x10^-10^  8.9 x10^-23^  7.3 x10^-11^  2.7 x10^-09^  8.5 x10^-10^  1.7 x10^-11^ | 0.22  0.87  0.38  0.03  0.61  0.92  0.19  0.50  0.66  0.50  0.54  0.06  0.30 | 2.80 x10^-13^  1.30 x10^-11^  3.68 x10^-10^  1.46 x10^-9^  N  N  0.09  0.16  N  N  N  0.04  N | N/A | Self-reported physical activity ~ 5%  Average acceleration 14% | 8.5 |
| Hara (2018) | Regular LTPA (a threshold of 4 MET-hrs/w) | rs10252228 | Intergenic region *NPSR1* and *DPY19L1* | 2.5 x 10^-8^ | 0.04 | 2.2x10^-9^ | *DNAPTP6* (rs12612420) meta-analysis p=0.0199  Not significant (p>0.05):  *PAPSS2*  *C18orf2*  *LEPR*  *GABRG3*  *RN7SK–SLC44A1* | 1.3%^b^ | 8 |
| De Moor (2009) | Regular exercisers vs non-exercisers using MET-hrs/w (a threshold of 4 MET-hrs/w) | rs238404  rs9789774  rs4355145  rs13013897  rs9633417  rs667923  rs1766581  rs10946904  rs10827786  rs2762527  rs12101846  rs12905612 | *ERCC2*  *C2orf3*  *C2orf3*  *SKIP*  *SGIP1*  *DNASE2B*  *SIPA1L2*  *PRSS16*  *ZNF248*  *PAPSS2*  *RORA*  *Q6UXP9_HUMAN* | 8.8x10^-5^  0.005  0.009  0.002  0.003  0.006  0.009  0.008  0.002  0.005  0.003  0.004 | 0.003  0.0004  0.003  0.0002  0.003  0.005  0.009  0.004  0.008  0.002  0.008  0.004 | 4.5x10^-6^  2.9x10^-5^  0.0003  6.1x10^-6^  0.0001  0.0003  0.0008  0.0004  0.0002  0.0001  0.0003  0.0002 | *LEPR* (rs12405556) p=0.0005, American sample  *CYP19A1*- (rs2470158) p=0.007, Dutch sample  Not significant (p>0.01)  *ACE, CASR, DRD2, MC4R* | N/R | 7.5 |
| Kim (2014) | Average daily physical activity level (METs) | rs7023003 | *RN7SK, SLC44A1* | 4.67x10^-6^ | N/A |  | N/A | N/R | 7 |
| Lin (2018) | Total LTPA-related energy expenditure (calculated as METs) | African- Americans:  rs116550874  rs3792874^c^  European Americans:  rs28524846 | *RERE, ENO1, ENO1-AS1, CA6, SLC2A7,*  *SLC2A5, GPR157*  *ACSL6,*  *LOC101927693,*  *IL3 ,CSF2, P4HA2,*  *LOC101927705,*  *PDLIM4,*  *SLC22A4,*  *LOC553103,*  *SLC22A5,*  *C5orf56,*  *LOC101927732,*  *IRF1, IL5,*  *RAD50*  *GPHN,*  *FAM71D,*  *MPP5,*  *ATP6V1D,*  *EIF2S1, PLEK2,*  *TMEM229B,*  *PIGH,*  *LOC100419668,*  *ARG2* | 9.92x10^-7^    6.32x10^-6^  9.10x10^-6^ | 4.20x10^-2^  3.95x10^-2^  6.16x10^-3^ | 1.63x10^-7^  8.33x10^-7^  1.30x10^-6^ | p<0.005  *GABRG3 (*rs72707657, rs12438610, rs12902711, rs12595253)*, CYP19A1* (rs62020072)*, PAPSS2* (rs1819162)*, CASR* (rs7650960, rs112909877, rs146555373, rs55716378)  Not significant p>0.005  *LEPR, DRD2, ACE, MC4R* | N/R | 7 |

Abbreviations: hrs, hours; LTPA, leisure time physical activity; MET, metabolic equivalent; N/A, not applicable; N/R, not reported; SNP, single nucleotide polymorphism; w, week

^a^ Combined p-value for cohort and replication sample (meta-analysis)

^b^ Observed-scale heritability

^c^ At this locus, three other SNPs around the lead SNP also have a p-value <1x10^-5^
