# Supplementary material for: Genetic variants related to physical activity or sedentary behaviour: a systematic review
Source: Int J Behav Nutr Phys Act. 2021 Jan 22;18:15. doi: 10.1186/s12966-020-01077-5 (PMC7821484; doi:10.1186/s12966-020-01077-5)
Supplement: Supplementary file 5 — Additional file 5. Overview of associations between candidate genes and physical activity or sedentary behavior in all included candidate gene studies. [file 12966_2020_1077_MOESM5_ESM.docx]

**Online supplementary 5**

Associations between candidate genes and physical activity or sedentary behaviour. Studies sorted in descending order according to quality score (high to low).

| **1^st^ author (year)** | **Gene (variant)** | **Phenotype** | **Association** | **95% CI** | **p-value** | **Quality score (0-12)** |
| --- | --- | --- | --- | --- | --- | --- |
| Bruneau (2017) | *ACE* (rs4340) | Walking distance (km/w) | Mean ± SD  Normal weight:  II: 15.8 ± 11.1  ID: 13.2 ± 10.6  DD: 17.9 ± 13.0  Overweight:  II: 16.7 ± 12.6  ID: 13.8 ± 11.6  DD: 9.7 ± 9.0 |  | II vs ID: 0.03  DD vs ID: 0.01  DD vs II: 0.02 | 9.5 |
| Bruneau (2018) | *IL15RA* (rs2228059) | Light intensity physical activity (hrs/w) | Mean ± SEM  AA: 39.4 ±2.4  CC: 28.6 ± 2.3 |  | 0.009 | 9.5 |
| Gielen (2014) | *PPARD*  (rs2267668)  (rs2076168) | Physical activity (counts/d) | AG vs AA  CC vs AA |  | 0.005  0.006 | 8.5 |
|  | *PPARGC1A* (rs8192678) | Vigorous physical activity (min/d) | AA vs GG |  | 0.001 |  |
| Murakami (2014) | *LEPR* (rs1137101) | Light physical activity (min/d) | Adjusted mean ± SD  RR: 559.4 ± 102.9  QQ/QR: 579.9 ± 103.1 |  | <0.05 | 8.5 |
|  |  | Inactive time (min/d) | Adjusted mean ± SD  RR: 815.5 ± 107.5  QQ/QR: 792.3 ± 107.7 |  | <0.05 |  |
| Camps (2019) | *FTO* (rs9939609) | Total activity (counts/d) | Mean  AT: 1.71  TT: 1.50  AA: 1.56 | 1.62-1.81  1.40- 1.59  1.39-1.74 | AT vs TT 0.008 | 8 |
| Maestu (2013) | *ACE* (in/del of 287-bp Alu repeat in intron 16) | Sedentary behaviour (min/d) | Mean ± SD  II: 403 ±79  ID: 412 ±74  DD: 391 ±52  I allele: 409 ±75  D allele: 403 ±66 |  | ID vs DD  <0.05    I allele vs DD <0.05 | 8 |
|  |  | Light physical activity (min/day) | Mean ± SD  II: 300 ±68  ID: 316 ±56  DD: 321 ±59  I allele:310 ±61  D allele: 318 ±57 |  | DD vs II <0.05  D allele vs II <0.05 |  |
|  |  | Total physical activity (min/day) | Mean ± SD  II: 354 ±9  ID: 375 ±7  DD: 382 ±8  I allele: 367 ±5  D allele 378 ±5 |  | DD vs II <0.05  D allele vs II <0.05 |  |
| Cole (2010) | *MC4R* (SNP1704) | Total activity (counts/d) | N/R |  | 0.004 | 7.5 |
|  | *MC4R* (SNP1704) | Time in moderate activity (%) | N/R |  | 0.016 |  |
|  | *MC4R* (SNP622) | Time in vigorous activity (%) | N/R |  | 0.021 |  |
| Wong (2012) | *ACE* (in/del of 287-bp Alu repeat in intron 16) | Low physical activity level | II: adjusted OR= 1.00  ID: adjusted OR= 4.34  DD: adjusted OR= 46.83  ID/DD: adjusted OR= 6.88 | ref  1.33-14.13  7.89-277.87  2.26-20.94 |  | 7.5 |
|  |  | Physical activity level (kcal/week) | Crude geometric mean  DD: 566  ID: 1160  II: 1798 |  | 0.0014 |  |
| Espinosa-Salinas (2019) | *GCKR* (rs780094) | Any physical activity at least once a week vs none | OR= 1.86  Minor allele greater frequency than major homozygous carriers | 1.36-2.56 | 0.004 | 7 |
| Flack (2019) | *CNR1* (rs6454672) | Moderate-to-vigorous physical activity (min/d) | Geometric mean ± SE  TT: 42.95 (2.48)  CT/CC 31.1 (2.1) |  | <0.01 | 7 |
| Goleva-Fjellet (2020) | *ACTN3 R577X* polymorphism | Low/medium vs high physical activity | X allele frequency 10% lower in high physical activity group |  | 0.006 | 7 |
| Lee (2015) | *MC4R* (rs17782313) | MET-hrs/w | C allele 3.5% less MET hour/week than TT |  | 0.02 | 7 |
|  | *TMEM18* (rs6548238) | MET-hrs/w | T allele 4.1% less than CC |  | 0.01 |  |
| Many (2017) | *ADRB1* (rs1801253) | Moderate physical activity (kcal/w) | Adjusted mean ± SEM  CC: 3314.2 ± 42.4  CG/GG: 2141.3 ± 69.7 |  | 0.040 | 7 |
| Reddon (2016) | *NTRK2* (rs1211166) | Physical activity level (sedentary, moderate active or very active) | OR 1.07 | 1.01-1.13 | 0.02 | 7 |
|  | *BDNF* (rs1401635) | Physical activity level (sedentary, moderate active or very active) | OR 1.08 | 1.03-1.14 | 2.7×10^−3^ |  |
|  | *NPC1* (rs1805081) | Physical activity level (sedentary, moderate active or very active) | OR 0.92 | 0.88-0.97 | 6.2×10^−4^ |  |
| Van Deveire (2012) | *ANKRD6* (rs61739327) | Moderate intensity physical activity (hrs/w) | Adjusted mean ± SEM  PP: 20.8 ± 0.7  PL: 20.7 ± 1.2  LL: 33.4 ± 4.7 |  | 0.03 | 7 |
| Lorentzon (2001) | *CASR* (A986S polymorphism) | Weight bearing physical activity (hrs/w) | Crude mean ± SD  AA: 4.3 ± 2.6  SS/AS: 2.9 ± 2.6 |  | 0.01 | 6.5 |
| Moleres (2009) | *IL-6* (rs1800795) | Leisure time physical activity (no activities/w, one activity/w, and more than one activity/w) | Crude mean ± SD  GG: 0.86 ± 0.70  GC/CC: 1.04 ± 0.79 |  | 0.012 | 6.5 |
| Richert (2007) | *LEPR* (Gln223Arg polymorphism) | Energy expenditure on physical activity (kcal/d) | Crude mean ± SEM  GG: 240.5 ± 11.8  GA: 266.6 ± 14.3  AA: 180.6 ± 21.0 |  | 0.016 | 6.5 |
| Walsh (2012) | *LEP19* (rs2167270) | Vigorous physical activity (kcal/w) | Adjusted mean ± SEM  GG: 1273.3 ± 176.8  GA/AA: 718.0 ± 147.2 |  | 0.02 | 6.5 |
|  |  | Leisure time sports activity (kcal/w) | Adjusted mean ± SEM  CG: 1922.8 ± 226.0  GA/AA: 1328.6 ± 188.2 |  | 0.04 |  |
|  |  | Light intensity physical activity (hrs/w) | Adjusted mean ± SEM  CG: 39.7 ± 1.6  GA/AA: 35.0 ± 1.4 |  | 0.03 |  |
| Klimentidis (2016) | *FTO* (rs9939609) | Sitting (hrs/d) | Meta-analysis  A allele:  Beta coefficient 0.11 |  | 2.3x10^-4^ | 6 |
| Loos (2005) | *MC4R* (rs7242169) | Moderate to vigorous exercise (total daily activity level based on METs/3-d)  Inactivity (total daily activity level based on METs/3-d) | Mean ± SEM  C/C: 223 ±20  C/T:200 ±20  T/T: 165 ±24  C/C: 456 ±8  C/T: 462 ±7  T/T 487 ±11 |  | 0.005  0.01 | 6 |
| Luglio (2016) | *UCP2*  (SNPs in UCP2  -866G/A) | Physical activity (METs/6-d) | Mean ± SD  A/A: 1850±106  G/A: 1945±129  G/G: 1962±129 |  | 0.02 | 6 |
| Murakami (2017) | *DRD2/ANKK1* (rs1800497) | Exercise habits from childhood to adolescence (regular exercises vs non-exercisers) | OR = 1.38  Increased likelihood to exercise for those carrying the TT allele | 1.06-1.80 | <0.05 | 5.5 |
| Wilkinson (2013) | *SNAP25* (rs363035) | Meeting physical activity recommendations (≥60 min/d on at least 5 of 7 days) | AA/AG vs GG:  Adjusted OR = 0.53 | 0.34-0.83 | 0.005 | 5.5 |
|  | *CNR1* (rs6454672) | Meeting physical activity recommendations (≥60 min/d on at least 5 of 7 days) | AA/AG vs GG:  Adjusted OR = 0.62 | 0.41-0.93 | 0.022 |  |
|  | *TPH2* (rs11615016) | Meeting physical activity recommendations (≥60 min/d on at least 5 of 7 days) | AA/AG vs GG:  Adjusted OR = 1.73 | 1.09-2.75 | 0.021 |  |
|  | *ACE* (rs8066276) | Meeting physical activity recommendations (≥60 min/d on at least 5 of 7 days) | AA/AG vs GG:  Adjusted OR = 1.44 | 1.08-1.91 | 0.012 |  |
| Simonen (2003) | *DRD2 (*polymorphism in exon 6 of the DRD2 gene) | Time spent in physical activity (hrs/w, men and women) | TT 22% lower than CT and 9% lower than CC |  | 0.020 | 5 |
|  |  | Total daily activity level based on METs/3-d (women) | Adjusted mean ± SEM  CC 1.24 ± 0.03  CT 1.33 ± 0.06  TT 0.99 ± 0.12 |  | 0.016 |  |
|  |  | Sports index (intensity, hrs/w, months/y), white women, replication cohort | Adjusted mean ± SEM  CC 1.65 ± 0.06  CT 1.77 ± 0.10  TT 1.28 ± 0.15 |  | 0.023 |  |
| Winnicki (2004) | *ACE* (in/del of 287-bp Alu repeat in intron 16) | Sedentary lifestyle | More sedentary in DD than II; 76% in DD, and  48% in II |  | 0.001 | 5 |
|  |  | Engaging in sports activities | % with allele genotype II:  13.7 in inactive  26.6 in active |  | 0.028 |  |
|  |  | Engaging in sports activities | % with allele genotype DD:  32.6 in inactive  18.0 in active |  | 0.032 |  |
| Grady (2013) | *DRD4* (VNTR polymorphism in exon 3) | Physical activity level (hrs/d in 1981) | 1.42 vs 0.98 hrs in DRD4 7R allele carriers vs non-carriers |  | 0.018 | 4 |
|  |  | Exercising ≥2 hrs/d (in 1981) | Twice as likely in DRD4 R7 vs non-carriers |  | 1.64x10^-5^ |  |
|  |  | Physical activity level (hrs/d, at the time of the study) | N/R |  | 0.033 |  |
| Salmen (2003) | *CYP19* (intron 4 TTTA repeat polymorphism) | Physical activity (hrs/w) | Crude percent with ≥3 hrs/week  Short allele repeats: 36.6  Mid allele repeats: 25.7  Long allele repeats: 19.6 |  | 0.039 | 4 |
| Good (2015) | *MAOA* (promoter VNTR polymorphism) | Physical activity level (mean score on 7-point Likert scale) | MAO-A VNTR genotype leading to high transcriptional activity showed lower physical activity |  | 0.047 | 1 |

Abbreviations: d, day; hrs, hours; in/del, insertion/deletion; MET, metabolic equivalent: OR, odds ratio; VNTR, variable number tandem repeat; w, week;

y, year
