# Supplementary material for: Genetic variants related to physical activity or sedentary behaviour: a systematic review
Source: Int J Behav Nutr Phys Act. 2021 Jan 22;18:15. doi: 10.1186/s12966-020-01077-5 (PMC7821484; doi:10.1186/s12966-020-01077-5)
Supplement: Supplementary file 6 — Additional file 6. Effect size, standard error and p-value for genetic variants from candidate genes studies associated with accelerometry defined phenotypes reported in summary statistics from the high quality GWAS. [file 12966_2020_1077_MOESM6_ESM.docx]

**Online supplementary 6**

Genetic variants identified in medium and high quality candidate gene studies that also were available in the summary statistics released from the GWAS by Doherty el al.^a^. Effect estimates and p-values are taken from the summary statistics by Doherty et al., and the table is sorted according to chromosome position. GWAS that identified candidate genes are indicated by grey cells. Estimates are only reported when a similar phenotype was available (overall activity, moderate activity, walking and sedentary).

| **Chromosome (position)** | **Gene (variant)^b^** | **Study reporting this association** | | **GWAS^a^** | | | **Quality score**  **(0-12)** |
| --- | --- | --- | --- | --- | --- | --- | --- |
|  |  | **1^st^ author (year)** | **Phenotype** | **Beta (SE)** | **p-value** | **Phenotype** |  |
| 1p31.3 | *LEPR* (rs1137101) | Murakami (2014) | Light physical activity (min/d) |  |  | No matching phenotype | 8.5 |
|  |  | Murakami (2014) | Inactive time (min/d) | -0.001 (0.005) | 0.76 | Sedentary | 8.5 |
| 1p31.3 | *LEPR* (rs12405556) | De Moor (2009) | Regular exercisers vs non-exercisers using MET-hrs/w (a threshold of 4 MET-hrs/w) | -0.002 (0.005) | 0.70 | Moderate activity | 7.5 |
| 1p31.3 | *LEPR* (Gln223Arg polymorphism, rs1137101^c^) | Richert (2007) | Energy expenditure on physical activity (kcal/d) | -0.001 (0.004) | 0.73 | Overall activity | 6.5 |
| 2p25.3 | *TMEM18* (rs6548238) | Lee (2015) | MET-hrs/w | -0.009 (0.006) | 0.11 | Overall activity | 7 |
| 2p23.3 | *GCKR* (rs780094) | Espinosa-Salinas (2019) | Any physical activity at least once a week vs none | -0.002 (0.005) | 0.64 | Overall activity | 7 |
| 2q33.1 | *DNAPTP6* (rs12612420) | Hara (2018) | Regular LTPA (a threshold of 4 MET-hrs/w) | 0.001 (0.006) | 0.81 | Overall activity | 8 |
| 3q13.3-q21.1 | *CASR*  (rs7650960, rs112909877, rs146555373, rs55716378) | Lin (2018) | Total LTPA-related energy expenditure (calculated as METs) | -0.008 (0.005)  -0.008 (0.005)  -0.009 (0.007)  -0.002 (0.006) | 0.12  0.14  0.22  0.79 | Overall activity | 7 |
| 3q13.3-q21.1 | *CASR* (A986S polymorphism, rs1801725^c^) | Lorentzon (2001) | Weight bearing physical activity (hrs/w) | -0.001 (0.007) | 0.88 | Overall activity | 6.5 |
| 4p15.2 | *PPARGC1A* (rs8192678) | Gielen (2014) | Vigorous physical activity (min/d) |  |  | No matching phenotype | 8.5 |
| 6p21.31 | *PPARD*  (rs2267668)  (rs2076168) | Gielen (2014) | Physical activity (counts/d) | -0.005 (0.006)  -0.004 (0.005) | 0.37  0.44 | Overall activity | 8.5 |
| 6q15 | *CNR1* (rs6454672) | Flack (2019) | Moderate-to-vigorous physical activity (min/d) | -0.003 (0.007) | 0.66 | Moderate activity | 7 |
| 6q15 | *ANKRD6* (rs61739327) | Van Deveire (2012) | Moderate intensity physical activity (hrs/w) | -0.0002 (0.007) | 0.98 | Moderate activity | 7 |
| 7p15.3 | *IL-6* (rs1800795) | Moleres (2009) | Leisure time physical activity (no activities/w, one activity/w, and more than one activity/w) | 0.002 (0.004) | 0.70 | Overall activity | 6.5 |
| 7q32.1 | *LEP19* (rs2167270) | Walsh (2012) | Vigorous physical activity (kcal/w) |  |  | No matching phenotype | 6.5 |
|  |  | Walsh (2012) | Leisure time sports activity (kcal/w) |  |  | No matching phenotype | 6.5 |
|  |  | Walsh (2012) | Light intensity physical activity (hrs/w) |  |  | No matching phenotype | 6.5 |
| 9q21.33 | *NTRK2* (rs1211166) | Reddon (2016) | Physical activity level (sedentary, moderate active or very active) | -0.007 (0.005) | 0.13 | Overall activity | 7 |
| 10p15.1 | *IL-15RA* (rs2228059) | Bruneau (2018) | Light intensity physical activity (hrs/w) |  |  | No matching phenotype | 9.5 |
| 10q23.2-q23.3 | *PAPSS2* (rs1819162) | Lin (2018) | Total LTPA-related energy expenditure (calculated as METs) | 0.004 (0.005) | 0.45 | Overall activity | 7 |
| 10q25.3 | *ADRB1* (rs1801253) | Many (2017) | Moderate physical activity (kcal/w) | -0.006 (0.005) | 0.29 | Moderate activity | 7 |
| 11p14.1 | *BDNF* (rs1401635) | Reddon (2016) | Physical activity level (sedentary, moderate active or very active) | -0.007 (0.005) | 0.13 | Overall activity | 7 |
| 11q13.2 | *ACTN3 R577X* polymorphism | Goleva-Fjellet (2020) | Low/medium vs high physical activity |  |  | No matching phenotype | 7 |
| 11q13.4 | *UCP2*  (SNPs in UCP2  -866G/A, rs659366^c^) | Luglio (2016) | Physical activity (METs/6-d) | -0.004 (0.005) | 0.43 | Overall activity | 6 |
| 15q12 | *GABRG3 (*rs72707657, rs12438610, rs12902711, rs12595253) | Lin (2018) | Total LTPA-related energy expenditure (calculated as METs) | -0.007 (0.010)  N/R  0.002 (0.005)  -0.005 (0.009) | 0.49 N/R  0.63  0.59 | Overall activity | 7 |
| 15q21.2 | *CYP19A1* (rs2470158) | De Moor (2009) | Regular exercisers vs non-exercisers using MET-hrs/w (a threshold of 4 MET-hrs/w | 0.0017 (0.007) | 0.83 | Overall activity | 7.5 |
| 15q21.2 | *CYP19A1* (rs62020072) | Lin (2018) | Total LTPA-related energy expenditure (calculated as METs) | -0.009 (0.006) | 0.12 | Overall activity | 7 |
| 16q12.2 | *FTO* (rs9939609) | Camps (2019) | Total activity (counts/d) | 0.001 (0.005) | 0.91 | Overall activity | 8 |
| 16q12.2 | *FTO* (rs9939609) | Klimentidis (2016) | Sitting (hrs/d) | -0.010 (0.005) | 0.04 | Sedentary | 6 |
| 17q23.3 | *ACE* (rs4340) | Bruneau (2017) | Walking distance (km/w) | N/R |  |  | 9.5 |
| 17q23.3 | *ACE* (in/del of 287-bp Alu repeat in intron 16,  rs4646994,  rs1799752,  rs4340,  rs13447447^c^) | Maestu (2013) | Sedentary behaviour (min/d) | N/R |  | Sedentary | 8 |
|  |  | Maestu (2013) | Light physical activity (min/day) |  |  | No matching phenotype | 8 |
|  |  | Maestu (2013) | Total physical activity (min/day) | N/R |  | Overall activity | 8 |
| 17q23.3 | *ACE* (in/del of 287-bp Alu repeat in intron 16,  rs4646994,  rs1799752,  rs4340,  rs13447447^c^) | Wong (2012) | Low physical activity level | N/R |  | Sedentary | 7.5 |
|  |  | Wong (2012) | Physical activity level (kcal/week) | N/R |  | Overall activity | 7.5 |
| 18q11.2 | *NPC1* (rs1805081) | Reddon (2016) | Physical activity level (sedentary, moderate active or very active) | -0.014 (0.005) | 0.002 | Overall activity | 7 |
| 18q21.32 | *MC4R* (SNP1704) | Cole (2010) | Total activity (counts/d) | Reference SNP ID not identified |  |  | 7.5 |
| 18q21.32 | *MC4R* (SNP1704) | Cole (2010) | Time in moderate activity (%) | Reference SNP ID not identified |  |  | 7.5 |
| 18q21.32 | *MC4R* (SNP622) | Cole (2010) | Time in vigorous activity (%) |  |  | No matching phenotype | 7.5 |
| 18q21.32 | *MC4R* (rs17782313) | Lee (2015) | MET-hrs/w | -0.002 (0.005) | 0.72 | Overall activity | 7 |
| 18q21.32 | *MC4R* (rs7242169) | Loos (2005) | Moderate to vigorous exercise (total daily activity level based on METs/3-d) | 0.001 (0.006) | 0.88 | Moderate activity | 6 |
| 18q21.32 | *MC4R* (rs7242169) | Loos (2005) | Inactivity (total daily activity level based on METs/3-d) | 0.007 (0.006) | 0.21 | Sedentary | 6 |

Abbreviations: d, day; hrs, hours; in/del, insertion/deletion; LTPA, leisure time physical activity; MET, metabolic equivalent; N/R, not reported; OR, odds ratio; SNP, single nucleotide polymorphism; VNTR, variable number tandem repeat; w, week;

y, year

^a^ Doherty, Aiden. Summary Statistics Relating to "GWAS Identifies 14 Loci for Device-Measured Physical Activity and Sleep Duration". University of Oxford, 2018

^b^ For GWAS genes reported to be closest to the SNP is presented

^c^ Reference SNP IDs were not reported in the original paper, but were looked up by the authors in similar literature and SNPedia, and cross-checked in dbSNP and the UCSC genome browser
